# Recombinant Human Growth Hormone and Rosiglitazone for Abdominal Fat Accumulation in HIV-Infected Patients with Insulin Resistance: A Randomized, Double-Blind, Placebo-Controlled, Factorial Trial

**DOI:** 10.1371/journal.pone.0061160

**Published:** 2013-04-12

**Authors:** Marshall J. Glesby, Jeanine Albu, Ya-Lin Chiu, Kirsis Ham, Ellen Engelson, Qing He, Varalakshmi Muthukrishnan, Henry N. Ginsberg, Daniel Donovan, Jerry Ernst, Martin Lesser, Donald P. Kotler

**Affiliations:** 1 Department of Medicine, Weill Cornell Medical College, New York, New York, United States of America; 2 Department of Public Health, Weill Cornell Medical College, New York, New York, United States of America; 3 Department of Medicine, St. Luke's-Roosevelt Hospital Center, New York, New York, United States of America; 4 Department of Medicine, Columbia University College of Physicians and Surgeons, New York, New York, United States of America; 5 AIDS Community Research Initiative of America, New York, New York, United States of America; 6 Feinstein Institute for Medical Research and Hofstra North Shore-LIJ School of Medicine, Manhasset, New York, United States of America; Pennington Biomedical Research Center, United States of America

## Abstract

**Background:**

Recombinant human growth hormone (rhGH) reduces visceral adipose tissue (VAT) volume in HIV-infected patients but can worsen glucose homeostasis and lipoatrophy. We aimed to determine if adding rosiglitazone to rhGH would abrogate the adverse effects of rhGH on insulin sensitivity (SI) and subcutaneous adipose tissue (SAT) volume.

**Methodology/Principal Findings:**

Randomized, double-blind, placebo-controlled, multicenter trial using a 2×2 factorial design in which HIV-infected subjects with abdominal obesity and insulin resistance were randomized to rhGH 3 mg daily, rosiglitazone 4 mg twice daily, combination rhGH + rosiglitazone, or double placebo (control) for 12 weeks. The primary endpoint was change in SI by frequently sampled intravenous glucose tolerance test from entry to week 12. Body composition was assessed by whole body magnetic resonance imaging (MRI) and dual Xray absorptiometry (DEXA).

Seventy-seven subjects were randomized of whom 72 initiated study drugs. Change in SI from entry to week 12 differed across the 4 arms by 1-way ANCOVA (P = 0.02); by pair-wise comparisons, only rhGH (decreasing SI; P = 0.03) differed significantly from control. Changes from entry to week 12 in fasting glucose and glucose area under the curve on 2-hour oral glucose tolerance test differed across arms (1-way ANCOVA P = 0.004), increasing in the rhGH arm relative to control. VAT decreased significantly in the rhGH arms (−17.5% in rhGH/rosiglitazone and −22.7% in rhGH) but not in the rosiglitazone alone (−2.5%) or control arms (−1.9%). SAT did not change significantly in any arm. DEXA results were consistent with the MRI data. There was no significant rhGH x rosiglitazone interaction for any body composition parameter.

**Conclusions/Significance:**

The addition of rosiglitazone abrogated the adverse effects of rhGH on insulin sensitivity and glucose tolerance while not significantly modifying the lowering effect of rhGH on VAT.

**Trial Registration:**

Clinicaltrials.gov NCT00130286

## Introduction

Altered fat distribution is prevalent among HIV-infected patients receiving antiretroviral therapy and can consist of loss of subcutaneous fat (lipoatrophy), gain of fat predominantly in the visceral depot (lipohypertrophy), or a combination of both [1], [2]. While lipoatrophy has been attributed to mitochondrial toxicity from thymidine analogue reverse transcriptase inhibitors [3], the pathogenesis of lipohypertrophy is poorly understood. Initiation of contemporary antiretroviral regimens that avoid thymidine analogues is associated with increases in visceral fat but uncommonly with loss of subcutaneous fat [4].

Some HIV-infected patients with altered fat distribution have decreased endogenous pulsatile growth hormone secretion that is inversely proportional to the amount of visceral fat [5], [6]. Administration of supraphysiologic doses of recombinant human growth hormone (rhGH) reduces visceral fat in HIV-infected patients with lipohypertrophy [7]–[10], but its use is limited by toxicities including adverse effects on glucose homeostasis. Although insulin resistance is common in HIV-infected patients with abdominal obesity, pivotal trials of rhGH excluded subjects with impaired glucose tolerance and diabetes mellitus [8], [9]. Though somewhat selective for visceral fat, administration of rhGH may also reduce subcutaneous fat modestly [8]–[10], which is an undesirable effect in patients with co-existing lipoatrophy.

Thiazolidinediones are peroxisome proliferator activator-γ agonists that improve insulin sensitivity and increase differentiation of pre-adipocytes into adipocytes [11]. While data are conflicting, rosiglitazone and pioglitazone may increase subcutaneous fat in HIV-infected patients who are not taking thymidine analogue drugs [12], [13]. We hypothesized that co-administration of rosiglitazone with rhGH to HIV-infected patients with lipohypertrophy and insulin resistance would abrogate the adverse effects of rhGH on insulin sensitivity and subcutaneous fat. To test this hypothesis, we conducted a randomized, double-blinded, placebo-controlled study of rhGH and rosiglitazone using a 2× factorial design.

## Methods

### Study Design

The protocol for this trial and supporting CONSORT checklist are available as supplementary information; see Protocol S1 and Checklist S1. This was a randomized, double-blind, placebo-controlled, multicenter trial using a 2×2 factorial design. Eligible subjects were randomized in a 1∶1:1∶1 ratio to receive recombinant human growth hormone (rhGH), rosiglitazone, rhGH + rosiglitazone, or double placebo treatment for 12 weeks.

The primary endpoint was the change in insulin sensitivity index (SI) from baseline to week 12 assessed by frequently sampled intravenous glucose tolerance test (FSIVGTT). Key secondary endpoints included changes from baseline to week 12 in visceral adipose tissue (VAT) and subcutaneous adipose tissue (SAT) volumes by magnetic resonance imaging (MRI) and total and regional fat mass by dual energy X-ray absorptiometry (DEXA).

### Participants

Eligible subjects were 18 to 65 years old with documented HIV-1 infection and on stable antiretroviral medications for at least 8 weeks. They had to meet established anthropometric criteria for excess abdominal fat consisting of both waist circumference >88.2 cm for men and 75.3 cm for women and waist∶hip ratio ≥0.95 cm for men and ≥0.90 for women [14], [15]. They also had to have evidence of insulin resistance based on a quantitative insulin sensitivity check index [16] (QUICKI)≤0.33. Subjects had to weigh >36 kg and have liver transaminase values ≤2.5 times the upper limit of normal, fasting glucose ≤6.94 mmol/L (125 mg/dL), two-hour glucose <11.1 mmol/L (200 mg/dL) on oral glucose tolerance test (OGTT), and fasting triglycerides ≤8.48 mmol/L (750 mg/dL). Subjects with active malignancy or systemic infection, prior diagnosis of pancreatitis, carpal tunnel syndrome, prior diagnosis of diabetes mellitus, coronary artery disease, disorder associated with moderate to severe edema, and untreated or uncontrolled hypertension, or contraindication to MRI were excluded. Subjects were permitted to use lipid lowering agents and testosterone therapy for hypogonadism (but no other androgenic/ anabolic agents) if started ≥12 weeks and ≥30 days prior to study entry, respectively. Race and ethnicity were self-reported by study subjects and categorized as per National Institutes of Health guidelines.

### Ethics

The study protocol was approved by the institutional review boards of all participating study sites (Weill Cornell Medical College Institutional Review Board, Columbia University Medical Center Institutional Review Board, St. Luke's-Roosevelt Institutional Review Board, and Western Institutional Review Board). All subjects provided written informed consent.

### Study Interventions and Assessments

Subjects self-administered rhGH 3 mg or matching saline placebo (Serostim®, EMD Serono, Rockland, MA) subcutaneously every evening and rosiglitazone (Avandia®, GlaxoSmithKline, Research Triangle Park, NC) 4 mg or matching placebo orally twice daily. The rhGH dose was based on demonstrated efficacy at reducing visceral fat in a previously published pilot study [17]. Subjects were asked to continue their usual diets and exercise routines throughout the study.

A 50% dose reduction of rhGH/placebo after initial interruption of treatment for up to 7 days was permitted to manage severe hyperglycemia (fasting glucose >7.77 mmol/L [140 mg/dL] or 2-hour OGTT glucose >13.3 mmol/L [240 mg/dL]), marked hypertension (symptomatic or ≥blood pressure 200/110 mmHg), fasting trigylcerides >20.3 mmol/L (1,800 mg/dL), or severe paresthesias. A 50% reduction of rhGH/placebo without interruption of treatment was permitted for moderate hyperglycemia (fasting glucose >6.99 mmol/L (126 mg/dL) and <7.77 mmol/L or 2-hour OGTT glucose >11.1 mmol/L (200 mg/dL) and <13.3 mmol/L, asymptomatic hypertension (blood pressure 140/90 to 200/110 mmHg), intolerable tissue turgor or arthralgias, fasting triglycerides 13.6 mmol/L (1200 mg/dL) to 20.30 mmol/L (1800 mg/dL),or symptoms of carpal tunnel syndrome. Sustained fasting glucose >6.99 mmol/L resulted in permanent discontinuation of rhGH/placebo. Subjects with peripheral edema were managed in a step-wise fashion: (1) counseled to reduce dietary sodium; (2) prescribed a low dose of diuretic; (3) reduced rhGH by 50%; (4) reduced rosiglitazone by 50%.

Study visits were scheduled at screening, pre-entry, entry, and weeks 2, 4, 6, 8, and 12 of the double-blind phase. Fasting labs and a two-hour OGTT were obtained at screening to determine eligibility. Anthropometric measurements were done in triplicate and averaged by trained staff using a Gulick II tape measure with tensiometer. Waist circumference was measured in the horizontal plane at a point immediately below the anterior part of the lowest ribs. Hip circumference was defined as the largest measurable circumference in the horizontal plane at a point between the iliac crest and greater trochanter. Eligible subjects underwent body composition testing in the fasted state on day 1 of entry and were admitted to one of three inpatient clinical research centers afterwards for performance of metabolic assessments the following morning. Body composition assessments consisted of whole body MRI and DEXA scanning as described previously [7] and measurement of total body and extracellular water volumes by isotope dilution. On day 2 of entry, subjects underwent a three hour insulin-modified FSIVGTT in the fasted state [18]. Samples for glucose and insulin determination were collected at −20, −15, −10, −5, 2, 3, 4, 5, 6, 8, 10, 12, 14, 16, 9, 22, 24, 25, 27, 30, 40, 50, 60, 90, 100, 120, 140, 160, and 180 minutes. At time 0, 300 mg/kg of glucose (50% dextrose) was administered by intravenous bolus followed by 0.03 U/kg of insulin at the 20 minute time point. Insulin sensitivity (SI) was calculated using MinMod software (Millennium, version 6.02, 3/29/04) [19]. Entry assessments were repeated at week 12. Subjects underwent two hour 75 g OGTTs at weeks 4 and 11 and routine safety laboratory testing at weeks 2, 4, 6, 8, and 12. Impaired fasting glucose was defined as a fasting glucose level of 5.6–6.9 mmol/L; impaired glucose tolerance as a 2-hour glucose value of 7.8–11.0 mmol/L; and, diabetes mellitus as a fasting glucose of ≥7.0 mmol/L or 2-hour value of ≥11.1 mmol/L. Forty-two subjects underwent assessments of basal lipolytic rate immediately prior to the FSIVGTT (data not reported herein).

Insulin and insulin were assayed in bulk on stored frozen samples at the New York Obesity Research Center Core Laboratory, St. Luke's-Roosevelt Hospital Center. Insulin was assayed using a commercial ^125^I labeling radioimmunoassay (Human insulin RIA kit, Linco Research, Inc. St. Charles, MO) and glucose was assayed using a Beckman Glucose Analyzer (Beckman Coulter, Fullerton, CA). The inter-assay coefficients of variation for assays of plasma insulin and glucose were 6% and <3%, respectively. Fasting lipids were measured by standard enzymatic methods. Serum concentration of IGF-1 was determined in the General Core Laboratory of the Weill Cornell Clinical and Translational Science Center using a quantitative sandwich enzyme immunoassay kit (R&D Systems, Inc., Minneapolis, MN) following the manufacturer's instructions. The intra-assay coefficient of variation is 3.5%–4.3%, and the inter-assay coefficient of variation is 7.5%–8.3%. The sensitivity of the assay is 0.026 ng/mL.

### Body Composition Methods

#### MRI

Whole body Magnetic Resonance Imaging (MRI) scans were performed using a 1.5 T system (Signa LX version 10; GE Medical Systems, Milwaukee) using a previously validated research protocol [7], [14]. Subjects lay supine with arms extended above their heads and were scanned in two segments with a common landmark at the L4–L5 inter-vertebral space. The spin-echo sequence had a 200 ms repetition time and a 14 ms echo time and acquired approximately 40 axial T1-weighted images with 10-mm thickness at 40-mm intervals from fingers to toes. The MRI images were analyzed by a single analyst (QH) using research image analysis software (SliceOmatic, version 4.0; Tomovision Inc, Montreal). Whole body adipose tissue was first segmented into visceral adipose tissue (VAT) and subcutaneous adipose tissue (SAT) compartments. Adipose tissue volumes were calculated according to the following formula: V = Σ(T+I)×S_i_, where T and I are slice thickness and inter-slice interval respectively, and S_i_ is the area of the tissue of interest on an individual image slice.

#### DEXA

Total body fat and total body fat-free mass were assessed using DEXA as previously described [7], [14]. The DEXA scanner (Lunar DPX, Lunar Radiation Corp., Madison WI) scans the whole body using an X-ray tube operating at two known X-ray energy levels. The scanner and system software calculate the attenuation of the different energy sources as they are absorbed by fat-free mass and lower density fat mass. Patients lay supine on the scanning table, positioned beneath the scanning arm, with transverse head-to-toe scans collected at 1.0 cm intervals.

#### Total Body Water (TBW) and Extracellular Water (ECW)

Assessment of TBW and ECW was determined by tracer dilution using deuterated water and sodium bromide, respectively. A baseline pre-tracer plasma sample was drawn followed by oral dosing of deuterium oxide and sodium bromide. An equilibrium plasma sample was obtained at steady state conditions three hours after dose administration. Samples were analyzed by infrared spectrophotometry to determine TBW [20] and liquid chromatography to determine ECW [21]. The reproducibility of the TBW and ECW tests are ±1% and ±1.3% respectively in the body composition laboratory.

### Sample Size

Since no pertinent data were available on SI by FSIVGTT with the interventions under study in this patient population, the sample size calculation was based on changes in insulin-stimulated glucose uptake by euglycemic hyperinsulinemic clamp in separate studies of HIV-infected subjects receiving rhGH 3 mg daily (data from week 4) [17] and rosiglitazone 4 mg twice daily (data from week 12) [22]. Details of the sample size calculation are provided in Appendix S1. Twenty subjects per arm with a premature discontinuation rate of 20% (i.e. 16 completers per arm) resulted in the following estimates of power: 39–99% for the rhGH main effect, 95–99% for the rosiglitazone main effect, and 5–94% for the interaction. We also performed an a priori power calculation for change in VAT at week 12 based on an estimated 1.25 L reduction in VAT with rhGH and −0.5 L reduction with rosiglitazone and no interaction. With 16 evaluable subjects per arm, we had 99% power for the rhGH main effect, 69% for rosiglitazone, and 69% for the interaction.

### Randomization—Sequence Generation

The randomization sequence was generated in SAS (SAS Institute Inc., Cary, NC) by a study statistician and provided to the research pharmacist. Randomization was stratified on study site and presence or absence of impaired glucose tolerance and used permuted blocks of four.

### Randomization—Allocation Concealment and Implementation

Study site personnel entered subject information into a customized, web-based electronic case report system, which generated sequential study numbers coded by study site and presence or absence of impaired glucose tolerance. The system generated a randomization sheet that was faxed to the central research pharmacy. The study pharmacist, who had no contact with the study subjects, determined the treatment assignment based on the randomization sequence and dispensed the appropriate study drugs. All treatment assignments were verified by a study statistician prior to dispensing study drugs. The allocation sequence was concealed from the researchers enrolling and assessing participants as only the central research pharmacist and statistician, neither of whom had contact with the participants, had access to the randomization list.

### Blinding

Study subjects and all study personnel were blinded to group assignments. Only the research pharmacist and study statistician had access to information about group assignment.

### Statistical Methods

Baseline data were summarized on all randomized subjects. Subjects who never initiated study drugs were excluded from safety and efficacy analyses. Given that the study focused on the physiologic effects of the interventions, analyses of changes from baseline to week 12 of metabolic or body composition parameters included only subjects with complete data at both time points; missing data were not imputed. Changes in dose or discontinuations of study drugs, however, were disregarded, and subjects were counted as if they continued on their randomized treatment as would be done in an intention to treat analysis. The mean and standard deviation (SD) for continuous variables were calculated at baseline and change at week 12 by study arm. Frequency and percentages are reported for categorical variables. The correlations between two continuous variables were calculated by Pearson correlation coefficient. The Jonckheere-Terpstra non-parametric test was used to test for differences between treatment arms and ordered categorical outcomes of glucose tolerance. One-way Analysis of Covariance (ANCOVA) was used to evaluate treatment effects on primary and secondary endpoints, and Dunnett-Hsu adjustment was used for post-hoc pair-wise comparisons to the double placebo arm. In addition, two-way ANCOVA was performed to evaluate the interaction of rosiglitazone and rhGH. We adjusted for the corresponding baseline values in all regression models with the endpoints of change at week 12 as outcomes. Normality of residuals from one-way and two-way ANCOVA were tested through the Kolmogorov-Smirnov test. If normality of residuals failed, the ANCOVA analysis was based on rank-transformed data [23], and median and first and third quartiles (Q1, Q3) of the original scale data were reported. The number of incident adverse events was tabulated using the maximum of relationship to treatment or severity for each subject. All p-values were two-sided, and p<0.05 was considered as statistical significance. Analyses were conducted with SAS software, version 9.2 (SAS Institute, Cary, NC).

The study was monitored by an independent Data and Safety Monitoring Board that reviewed accrual, data completeness, and adverse event data by blinded study arm. No efficacy data were reviewed, and no statistical testing was done for the interim safety reviews.

## Results

### Disposition of Subjects and Baseline Characteristics

**Figure 1:**
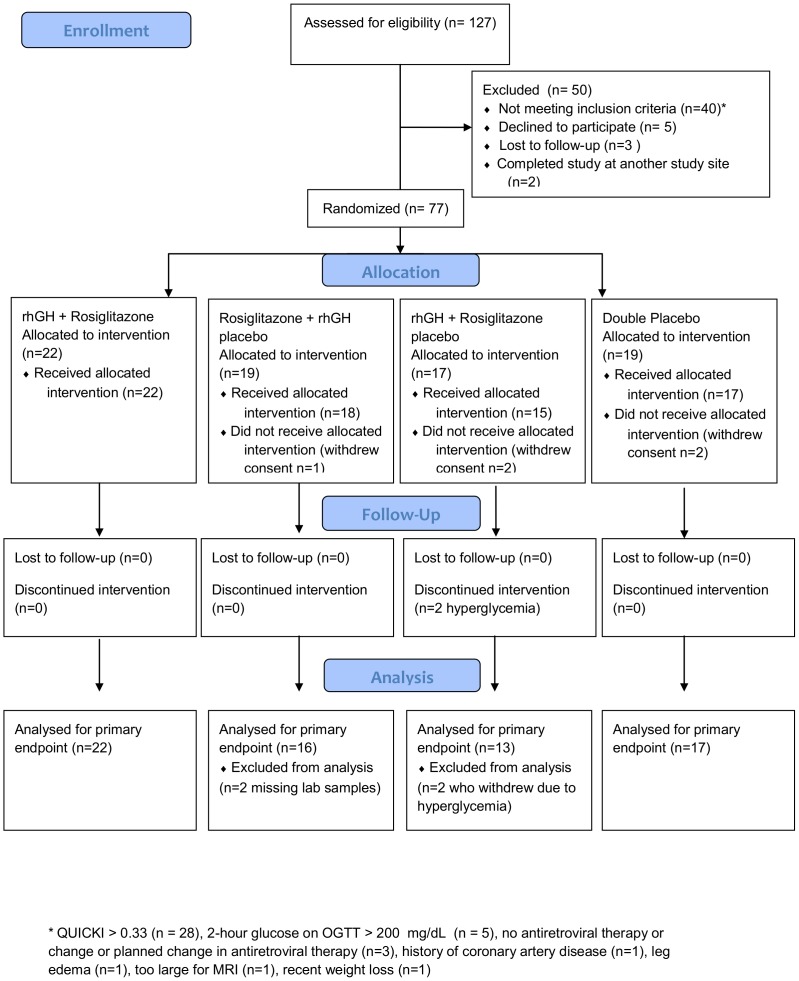
CONSORT Flow Diagram.

From February 2005 to December 2009, we screened 127 subjects, of whom 77 were randomized (Figure 1) [24]. The most common reasons for screen failure were QUICKI>0.33 (n = 6), withdrawal of consent or loss to follow-up (n = 7), and 2-hour glucose on OGTT>11.1 mmol/L (n = 5). Of the 77 randomized subjects, 5 withdrew consent prior to initiating study drug. Rates of completion of week 12 of the study were 22/22 on rosiglitazone/rhGH, 17/18 on rosiglitazone, 13/15 on rhGH, and 17/17 on double placebo.

Table 1 summarizes the baseline characteristics of all randomized subjects (n = 77); characteristics were similar when restricted to the 72 subjects who initiated study drugs Overall, the mean age was 47.9 (7.1), 77.9% were men, 31.2% were black, and 39.0% Hispanic. Most subjects were on protease inhibitor or non-nucleoside reverse transcriptase inhibitor based regimens, and 29.9% were receiving thymidine analogues. The mean CD4 count was 579 (255)×10^6^ cells/L and 66.2% had suppressed HIV viral loads. Use of testosterone supplementation, lipid-lowering therapy, and anti-hypertensives was common. Overall, the mean BMI was 29.1 (4.4) kg/m^2^, waist circumference 103.7 (9.88) cm, and waist∶hip ratio 1.04 (0.06).

**Table 1:** Baseline Characteristics.

Category	Variable	Variable Subcategory	rhGH/Rosiglitazone (n = 22)	Rosiglitazone (n = 19)	rhGH (n = 17)	Double Placebo (n = 19)
Demographic	Age, years		46.8 (9.4)	49.3 (6.1)	48.6 (4.9)	46.6 (6.7)
	Sex	Male	18 (81.8%)	15 (78.9%)	14 (82.4%)	13 (68.4%)
	Race	White	15 (68.2%)	11 (57.9%)	11 (64.7%)	12 (63.2%)
		Black	6 (27.3%)	6 (31.6%)	6 (35.3%)	6 (31.6%)
		>1 race	1 (4.6%)	2 (10.5%)	0	1 (5.3%)
	Ethnicity	Hispanic	9 (40.9%)	9 (47.4%)	3 (17.6%)	9 (47.4%)
		Non-Hispanic	13 (59.1%)	10 (52.6%)	14 (82.4%)	10 (52.6%)
HIV	Antiretro-viral regimen	≥2 NRTIs+NNRTI	7 (31.8%)	7 (36.8%)	8 (47.1%)	7 (36.8%)
		≥2 NRTIs+PI	11 (50%)	9 (47.4%)	6 (35.3%)	10 (52.6%)
		≥3 NRTIs	2 (9.1%)	0	2 (11.8%)	0
		Other	2 (9.1%)	3 (15.8%)	1 (5.9%)	2 (10.5%)
		Stavudine or zidovudine use	5 (22.7%)	5 (26.3%)	5 (29.4%)	8 (42.1%)
Concomitant Medications	Testosterone	6 (27.7%)	2 (10.5%)	5 (29.4%)	2 (10.5%)	
	Atypical antipsychotic	5 (22.7%)	3 (15.8%)	1 (5.9%)	0	
	Fibrate	3 (13.6%)	2 (10.5%)	3 (17.6%)	3 (15.8%)	
	Statin	7 (31.8%)	7 (36.8%)	5 (29.4%)	5 (26.3%)	
	Fish oil	0	1 (5.3%)	0	1 (5.3%)	
	Antihypertensive	6 (27.3%)	5 (26.3%)	5 (29.4%)	5 (26.3%)	
Anthropomet-ric	Height, cm	172 (11)	171 (8)	174 (9)	169 (8)	
	Weight, kg	89.4 (20.0)	78.6 (8.6)	92.0 (11.1)	83.3 (17.0)	
	Body mass index, kg/m^2^	30.1 (5.1)	26.9 (3.2)	30.3 (3.3)	28.9 (4.7)	
	Waist circumference, cm	105.13 (11.48)	99.69 (6.34)	107.48 (8.25)	102.73 (11.1)	
	Hip circumference, cm	100.22 (7.52)	96.27 (4.76)	103.46 (7.74)	99.4 (10.05)	
	Waist∶hip ratio	1.05 (0.06)	1.04 (0.06)	1.04 (0.05)	1.03 (0.06)	
Laboratory Values	Fasting insulin, pmol/L	140.4 (50.8)	171.9 (186.3)	152.2 (126.0)	132.0 (65.4)	
	Fasting glucose, mmol/L	5.05 (0.48)	4.77 (0.42)	5.30 (0.52)	5.18 (0.63)	
	QUICKI	0.31 (0.01)	0.31 (0.02)	0.31 (0.02)	0.31 (0.02)	
	CD4 count*, cells ×10^6^/L	555 (272)	595 (251)	56 (245)	617 (271)	
	HIV RNA<400** copies/ml	17 (77.3%)	14 (82.3%)	12 (75.0%)	8 (53.3%)	

Continuous variables are expressed as mean (standard deviation).

* CD4 count available on n = 20, 16, 14, and 13 in rhGH/rosiglitazone, rosiglitazone, rhGH, and double placebo arms respectively.

** HIV RNA data available on n = 22, 17, 16, and 15 in rhGH/rosiglitazone, rosiglitazone, rhGH, and double placebo arms respectively.

Abbreviations: NNRTI, non- nucleoside reverse transcriptase inhibitor; NRTI, nucleoside reverse transcriptase inhibitor; PI, protease inhibitor; QUICKI, quantitative insulin sensitivity check index.

### Adverse Events

Table 2 summarizes treatment emergent adverse events by study arm. The numbers of adverse events considered possibly/probably related to study drug(s)/placebo(s) were: 17 in the rosiglitazone/rhGH arm (6 resulting in dose reduction of rhGH and 0 in drug discontinuation); 11 in the rosiglitazone arm (2 resulting in dose reduction of rhGH placebo and 0 in drug discontinuation); 14 in the rhGH arm (4 resulting in dose reduction of rhGH and 2 in rhGH discontinuation); and 9 in the double placebo arm (none resulting in dose reduction or drug discontinuation). The most common adverse events were edema and musculoskeletal symptoms/signs, which were seen predominantly in the rhGH-containing arms.

**Table 2:** Treatment Emergent Adverse Events*.

Adverse Event Category	Adverse Event	rhGH+Rosiglitazone (n = 22)			Rosiglitazone (n = 18)			rhGH (n = 15)			Double placebo (n = 17)		
		Any grade	Grade 3	Grade 4	Any grade	Grade 3	Grade 4	Any grade	Grade 3	Grade 4	Any grade	Grade 3	Grade 4
Constitutional													
	Fever	0	0	0	1	0	0	1	0	0	2	0	1
	Fatigue	7	0	1	6	1	0	5	1	0	5	2	0
	Weight gain	1	0	0	0	0	0	1	0	0	2	0	0
	Headache	3	1	0	2	0	0	3	2	0	2	0	0
Musculoskeletal													
	Arthralgia	7	2	2	4	0	0	5	1	1	5	2	0
	Carpal tunnel syndrome	1	0	1	1	0	0	3	2	0	0	0	0
	Myalgia	6	3	2	4	2	0	6	4	0	3	1	0
	Paresthesias	5	1	0	1	1	0	4	0	0	2	0	0
	Back pain	4	2	0	4	1	0	4	2	0	2	1	0
Metabolic													
	Hypertriglycerid-emia	0	0	0	1	0	1	2	2	0	3	1	2
	Hyperglycemia	5	2	0	1	1	0	8	3	1	2	0	0
Hepatic													
	Increased ALT	2	0	0	2	0	0	0	0	0	0	0	0
	Increased AST	1	0	0	2	0	0	1	0	0	1	0	0
Gastrointestinal													
	Nausea	2	0	0	0	0	0	1	0	0	1	0	0
	Vomiting	0	0	0	1	0	0	0	0	0	3	0	0
	Abdominal pain	0	0	0	1	0	1	2	1	0	1	0	0
Other													
	Edema	12	1	1	3	1	0	11	3	0	2	0	0
	Breast enlargement/ten-derness	4	1	0	1	0	0	0	0	0	0	0	0
	Cough/upper respiratory infection	0	0	0	0	0	0	2	0	0	2	1	0

* Only adverse events seen in >5% of subjects are reported.

The cumulative incidence of fasting hyperglycemia reported in real time as an adverse event by study arm was: 5/22 (including two grade 3) in the rosiglitazone/rhGH arm, 1/18 (one grade 3) in the rosiglitazone arm, 8/15 (including three grade 3 and one grade 4) in the rhGH arm, and 2/17 (all<grade 3) in the double placebo arm. One subject in the rosiglitazone/rhGH arm dose reduced rhGH due to hyperglycemia; two in the rosiglitazone arm dose reduced rhGH placebo due to hyperglycemia; and two in the rhGH arm discontinued rhGH and one dose reduced rhGH due to hyperglycemia. There were no serious adverse events considered to be possibly, probably, or definitely related to study drugs.

### Insulin Sensitivity and Glucose Tolerance

By FSIVGTT, the change in SI from entry to week 12 differed significantly across the 4 arms by 1-way ANCOVA on rank-transformed data (P = 0.0002; Table 3); by pair-wise comparisons relative to the double placebo arm, SI decreased significantly in the rhGH arm (P = 0.02). There was no significant rhGH x rosiglitazone interaction for change in SI by 2-way ANCOVA (P = 0.97); pooling across arms, the rhGH main effect (decreasing SI; P<.0001) and rosiglitazone main effect (increasing SI; P = 0.003) were statistically significant.

**Table 3:** Baseline and Change at Week 12 in Glucose Homeostasis Parameters by Frequently Sampled Intravenous Glucose Tolerance Test and Oral Glucose Tolerance Test.

Variable	Rosiglitzone+rhGH		Rosiglitazone		rhGH		Double Placebo		P-value*
	Entry	Change at week 12	Entry	Change at week 12	Entry	Change at week 12	Entry	Change at week 12	
	N = 22	N = 22	N = 18	N = 16	N = 15	N = 13	N = 18	N = 17	
SI, μU * 10^−4^ * min^1^ * ml^−1^									
Median (Q1, Q3)	1.66 (0.95, 2.33)	0.20^a^ (−0.37, 1.00)	1.97 (1.41, 2.70)	1.44^b^ (0.68, 2.59)	1.72 (1.14, 3.64)	−0.63^c^ (−1.03, 0.11)	1.71 (1.47, 3.71)	0.14 (−0.41, 1.21)	0.0002
	N = 20	N = 20	N = 17	N = 15	N = 14	N = 13	N = 17	N = 14	
Fasting glucose, mmol/L Mean (SD)	5.21 (0.58)	0.28^d^ (1.15)	4.95 (0.50)	0.39^e^ (0.81)	5.56 (1.84)	0.86^f^ (1.88)	5.30 (0.47)	−0.11 (0.46)	0.026
	N = 19	N = 19	N = 17	N = 15	N = 14	N = 13	N = 17	N = 14	
Fasting insulin, pmol/L Median (Q1, Q3)	191.20 (140.84, 197.03)	23.26 (−50.91, 105.15)	118.20 (97.23, 164.67)	−23.75 (−67.92, 12.08)	132.72 (74.24, 279.19)	22.78 (−33.06, 90.35)	162.10 (104.45, 187.58)	2.22 (−28.96, 27.85)	0.15
	N = 20	N = 20	N = 17	N = 16	N = 14	N = 13	N = 17	N = 14	
Glucose AUC, mmol/L*2 h Mean (SD)	30.3 (5.23)	−0.060^g^ (6.22)	30.4 (8.11)	−0.87^h^ (5.11)	31.5 (8.10)	3.59^i^ (4.50)	30.7 (5.85)	−1.20 (4.25)	0.034
	N = 19	N = 19	N = 17	N = 16	N = 14	N = 13	N = 17	N = 14	
Insulin AUC, pmol/L*2h Mean (SD)	134,157 (105,133)	−17,793 (117,593)	125,635 (172,972)	−39,482 (138,282)	128,323 (118,634)	11,813 (100,550)	146,012 (121,197)	−17,460 (61,026)	0.33

Data are expressed as mean (standard deviation).

* One-way ANCOVA (done on rank-transformed data for SI and fasting insulin).

rhGH x rosiglitazone interaction term was only statistically significant for fasting glucose in two-way ANCOVA (P = 0.020).

a P = 0.69,

b P = 0.09,

c P = 0.02,

d P = 0.84,

e P = 0.91,

f P = 0.014,

g P = 0.88,

h P = 0.99,

i P = 0.026 compared to double placebo group.

Abbreviations: Q1 (1^st^ quartile), Q3 (3^rd^ quartile); SI, insulin sensitivity; AUC, area under the curve.

Change in fasting glucose from entry to week 12 differed across arms, driven by a statistically significant increase of 0.86 mmol/L in the rhGH alone arm (P = 0.014). The rosiglitazone x rhGH interaction for change in fasting glucose was statistically significant (P = 0.039). Fasting insulin and insulin area under the curve on OGTT did not change significantly across the study arms. Change in glucose area under the curve on OGTT from entry to week 12 differed across arms (1-way ANCOVA P = 0.004), increasing in the rhGH arm relative to double placebo (P = 0.026) but not differing in the other arms. By 2-way ANCOVA, the rhGH x rosiglitazone interaction was borderline significant (P = 0.094).

**Figure 2:**
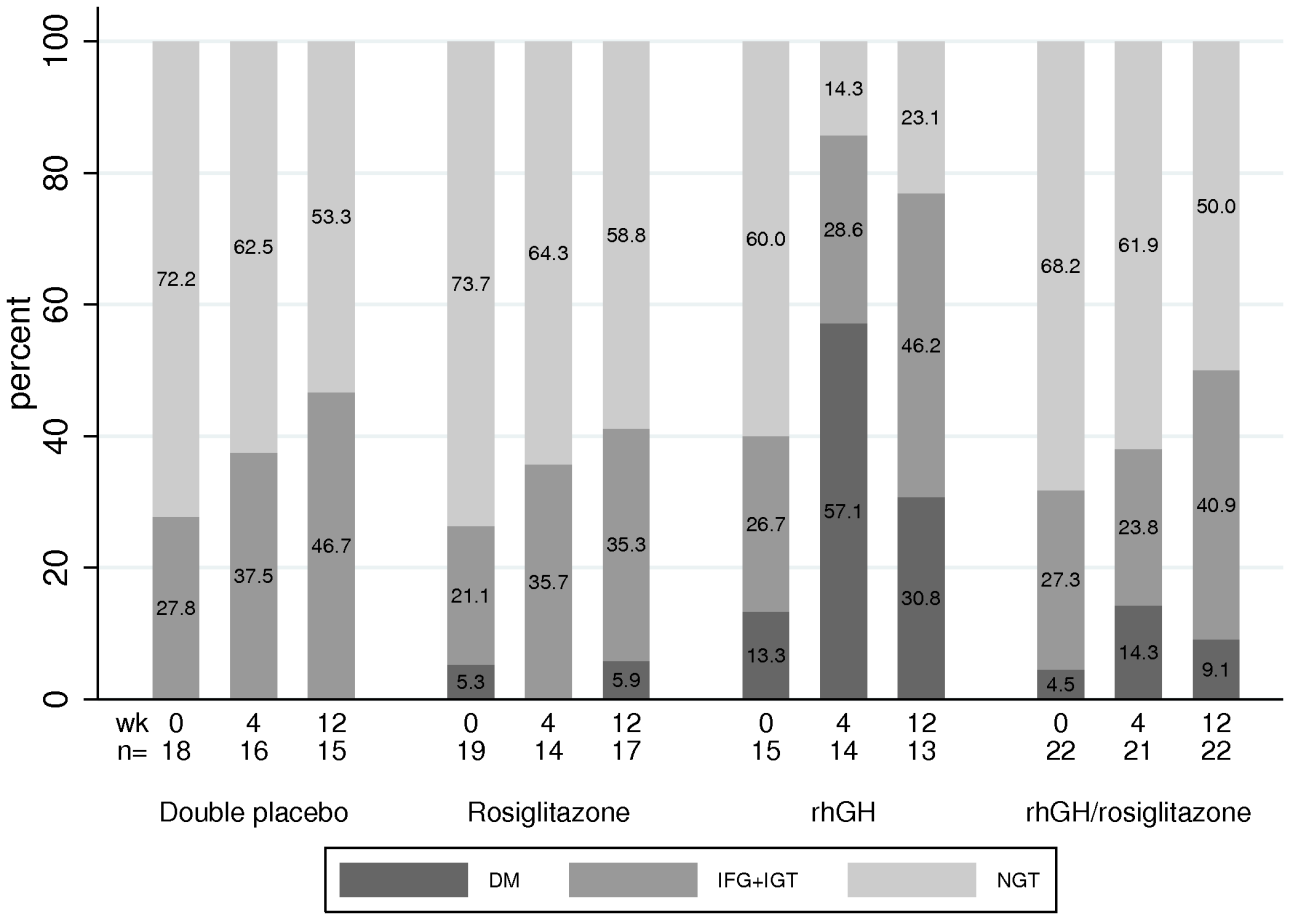
Changes in glucose homeostasis category by 2-hour oral glucose tolerance test. The trends for shifts in categories were not statistically different across study arms (P = 0.84, exact Jonckheere-Terpstra test). Abbreviations: DM, diabetes mellitus; IFG, impaired fasting glucose; IGT, impaired glucose tolerance; NGT, normal glucose tolerance.

Figure 2 summarizes changes in glucose tolerance categories over time by study arm. Six of 22 (27%) on rosiglitazone/rhGH, 3 of 17 (18%) on rosiglitazone alone, 6 of 13 (46%) on rhGH alone, and 4 of 15 (27%) on double placebo shifted OGTT categories adversely at week 12 (P = 0.84, exact Jonckheere-Terpstra test). Specifically, in the rosiglitazone/rhGH arm one subject with normal glucose tolerance at entry and one with impaired glucose tolerance were diabetic at week 12. In the rosiglitazone alone arm, one subject with normal glucose tolerance was diabetic at week 12. In the rhGH alone arm, two with normal glucose tolerance were diabetic at week 12. Of note, two other subjects in the rhGH arm who qualified for the study based on screening fasting glucose and 2-hour glucose values on OGTT done at the local laboratories in real time met criteria for diabetes mellitus retrospectively on batched testing of stored OGTT samples and remained classified as diabetic at week 12. Similarly, one subject each in the rosiglitazone/rhGH arm and the rosiglitazone arm was diabetic at entry and had impaired glucose tolerance at week 12. In the double placebo arm, no subjects were classified as diabetic at week 12.

### Body Composition

Table 4 summarizes changes in body composition by whole body MRI scan. VAT decreased significantly from baseline in the rosiglitazone/rhGH and rhGH arms compared to placebo (−17.5% and −22.7% versus −1.87%, respectively) but did not change significantly in the rosiglitazone arm (−2.5%). There was no significant rhGH x rosiglitazone interaction for change in VAT by 2-way ANCOVA (P = 0.70); pooling across arms, the rhGH main effect (decreasing VAT; P = 0.0001) but not rosiglitazone main effect (P = 0.69) was statistically significant. There were no statistically significant changes across the arms in SAT or TAT by one-way ANCOVA. Neither the interaction terms nor the main effects were statistically significant in the two-way ANCOVA models for SAT and TAT. The change in skeletal muscle volume was statistically significant across the four arms by 1-way ANCOVA (P = 0.014), with trends for modest increases in the rhGH-containing arms (rosiglitazone/rhGH 4.72% and rhGH 6.71%). By 2-way ANCOVA, the rhGH main effect on skeletal muscle volume was statistically significant (P = 0.0071) but not the rosiglitazone main effect (P = 0.68). Overall, change in VAT did not correlate with change in SI (r = 0.0097; P = 0.94).

**Table 4:** Baseline and Change at Week 12 in Body Composition Parameters by Whole Body MRI.

Variable	rhGH/Rosiglitazone		Rosiglitazone		rhGH		Double Placebo		P-value*
	Entry (n = 20)	Change at week 12 (n = 20)	Entry (n = 18)	Change at week 12 (n = 15)	Entry (n = 14)	Change at week 12 (n = 11)	Entry (n = 17)	Change at week 12 (n = 16)	
VAT, L	5.14 (2.74)	−1.13^a^ (1.41)	4.73 (2.01)	−0.19^b^ (0.69)	5.29 (2.29)	−1.15^c^ (0.81)	5.40 (1.88)	−0.04 (0.9)	0.0012
SAT, L	22.69 (8.59)	−0.11 (3.33)	20.98 (8.61)	0.74 (1.86)	26.41 (8.83)	−0.38 (1.23)	24.46 (9.52)	−0.03 (2.64)	0.89
TAT, L	31.99 (9.97)	−1.39 (4.08)	30.12 (6.33)	0.01 (1.49)	35.13 (7.97)	−0.93 (1.88)	32.20 (7.22)	−0.10 (3.25)	0.53
SM, L Median (Q1, Q3)	27.12 (22.82, 34.59)	0.70^e^ (−0.09, 1.55)	26.16 (23.10, 28.74)	−0.34^f^ (−1.17, 0.23)	30.36 (26.34, 33.95)	1.11^g^ (−0.46, 2.48)	25.49 (23.58, 31.04)	−0.27 (−1.29, 0.53)	0.014

Data are expressed as mean (standard deviation).

* One-way ANCOVA (done on rank-transformed data for SM). rhGH x rosiglitazone interaction term was not statistically significant for any variables in two-way ANCOVA.

a P = 0.003,

b P = 0.91,

c P = 0.01,

e P = 0.08,

f P = 0.99,

g P = 0.05 compared to double placebo group.

Abbreviations: L, liter. Q1 (1^st^ quartile), Q3 (3^rd^ quartile). rhGH, recombinant human growth hormone; VAT, visceral adipose tissue volume; SAT, subcutaneous adipose tissue volume; TAT, total adipose tissue volume; SM, skeletal muscle.

Data on changes in body composition by DEXA and stable isotope dilution are summarized in Table S1. Body weight did not change significantly across the study arms. Total and trunk fat tended to decrease in the rhGH-containing arms (−10.7% and −12.8% in the rosiglitazone/rhGH and −8.78% and −11.9% rhGH arms, respectively), though not achieving statistical significance from one-way ANCOVA. Lean body mass increased significantly in the rhGH-containing arms (5.97% and 4.58% in the rosiglitazone/rhGH and in the rhGH arms, respectively). Limb fat changed significantly across study arms (P = 0.026); pair-wise comparisons were statistically significant only for the comparison of rosiglitazone alone (+8.8%) to placebo (P = 0.044). By 2-way ANCOVA, the rhGH x rosiglitazone interaction was borderline significant (P = 0.069), suggestive of rhGH antagonizing the effect of rosiglitazone on increasing limb fat. Total body water and extracellular water volumes did not change significantly across study arms.

### Insulin-Like Growth Factor-1 (IGF-1)

Data on changes in IGF-1 from baseline to week 12 were available on a subset of subjects who completed week 12 evaluations and had available frozen serum. At baseline, IGF-1 concentrations were 107.3±37.7 ng/ml (n = 14) in the rhGH/rosiglitazone arm, 110.0±33.2 ng/ml (n = 12) in the rosiglitazone arm, 100.6±23.7 ng/ml (n = 9) in the rhGH arm, and 86.6±31.9 (n = 12) ng/ml in the double placebo arm. The changes at week 12 were 189.9% in the rhGH/rosiglitazone arm, −0.90% in the rosiglitazone arm, 123.6% in the rhGH arm, and 13.4% in the double placebo arm. By 1-way ANCOVA, these changes differed across arms (P<0.0001). Pair-wise comparisons yielded significant differences in the rhGH/rosiglitazone and rhGH arms relative to double placebo (P<0.001 and P = 0.020, respectively). The rhGH x rosiglitazone interaction term for change in IGF-1 by 2-way ANCOVA was not statistically significant (P = 0.13); pooling across arms, the rhGH main effect (increasing IGF-1; P<0.0001) but not rosiglitazone main effect (P = 0.44) was statistically significant.

### Lipids

Fasting triglycerides were monitored in real time as a safety measure (summarized in Table 2). Fasting total cholesterol, calculated low density lipoprotein-cholesterol, high density lipoprotein-cholesterol (HDL-C), triglycerides, and calculated non-HDL-C were determined on the subset of subjects who had available frozen serum at entry, week 4 and week 12. There were no statistically significant changes in lipid parameters over time by study arm (data not shown).

## Discussion

In this randomized, double-blind, placebo-controlled trial, HIV-infected subjects with abdominal obesity and insulin resistance received rhGH, rosiglitazone, dual therapy, or double placebo for 12 weeks. In keeping with our primary hypothesis, the addition of rosiglitazone to rhGH abrogated the adverse effects of rhGH on insulin sensitivity as assessed by FSIVGTT and OGTT without modifying the favorable effects of rhGH on reducing VAT. There were also fewer hyperglycemia events in the dual therapy arm compared to the rhGH alone arm.

Combination therapy was relatively well tolerated in this study. Despite the potential for rosiglitazone to increase plasma volume, we found no evidence that edema and musculoskeletal side effects attributable to fluid retention were exacerbated by the addition of rosiglitazone to rhGH. Furthermore, we were unable to detect significant changes in total body water or extracellular water volumes by isotope dilution methods across the study arms.

Studies to date suggest that growth hormone adversely affects insulin sensitivity primarily by stimulating lipolysis and increasing free fatty acid concentrations. Administration of acipimox, a nicotinic acid derivative that inhibits lipolysis at the level of hormone-sensitive lipase, to growth hormone-deficient adults receiving rhGH replacement abrogated the effects of rhGH on insulin sensitivity [25], [26]. Thiazolidinediones have pleiotropic effects that likely account for their favorable effects on insulin sensitivity; however, their effects on suppressing lipolysis and reducing free fatty acid concentrations may play a major role in this regard [27], [28]. These observations provided the biological rationale for combining rosiglitazone with rhGH in our study. Ongoing analyses of free fatty acid flux from a subset of our participants should shed further light on the physiologic effects of the combination of study drugs since data in humans are limited.

Our study is one of the first in HIV-infected subjects to combine rhGH with a drug aimed at improving insulin sensitivity. Within the context of a broader pilot study investigating multiple strategies to manage altered fat distribution in HIV-infected subjects, Macallan and colleagues randomly assigned 26 subjects with lipoatrophy, of whom about half had visceral adiposity, to receive rhGH 2 mg daily or rhGH 2 mg daily plus rosiglitazone 4 mg daily for 12 weeks [29]. Mean fasting plasma insulin increased and HOMA %S (insulin sensitivity; the reciprocal of HOMA-insulin resistance) decreased within the rhGH arm but did not change significantly from baseline within the dual therapy arm, suggesting a beneficial effect of the addition of rosiglitazone on glucose homeostasis. Of note, our study population differed from that of Macallan et al in that all of our subjects had visceral adiposity, the population for which manipulation of the GH axis could be beneficial, as well as insulin resistance.

We found that rosiglitazone did not interact with rhGH with regard to changes in body composition. The thiazolidinedione drugs have had inconsistent effects on reducing VAT in patients with diabetes mellitus in the general population [30]–[34]. In a randomized, double-blind, placebo-controlled trial with a 2×2 factorial design, pioglitazone and rhGH (8 µg/kg/d) were studied in 62 abdominally obese adults [35]. Pioglitazone alone did not reduce VAT significantly, whereas the combination of rhGH and pioglitazone reduced VAT similarly to rhGH alone. Studies of rosiglitazone or pioglitazone for lipoatrophy in HIV-infected patients, however, have generally not had favorable effects on VAT. The effects of rosiglitazone and pioglitazone on HIV-associated lipoatrophy have also been mixed [36], [37]. Patients who were not currently taking thymidine analog reverse transcriptase inhibitors did gain limb fat with rosiglitazone [13] and pioglitazone [12] in two studies. Our study subjects were not selected on the basis of having lipoatrophy, and most were not currently taking thymidine analogs. Nonetheless, we did not find significant changes in subcutaneous fat volume by MRI or arm or leg fat mass by DEXA in the rosiglitazone-containing arms relative to placebo.

Our study has several strengths, including the randomized, double-blind design, the use of sensitive, standardized physiological and body composition assessments, and the diverse patient population. The study limitations include the fact that we did not fully enroll the study and may have been underpowered for the primary endpoint despite excellent retention of subjects. Due to slower than expected accrual, there may have been temporal changes in antiretroviral therapy during the course of the study, though individual subjects did not change therapy during the blinded phase of the study. The randomized design, however, makes it unlikely that inter-arm differences were a source of bias. Lastly, our study population included subjects who met an arbitrary definition of insulin resistance, and the results cannot be generalized to patients with frank diabetes or who do not have insulin resistance.

Both study drugs have fallen out of favor since the design and conduct of our study. Growth hormone did not receive approval by the U.S. Food and Drug Administration (FDA) for the indication of reducing abdominal fat in HIV-infected subjects despite efficacy demonstrated in clinical trials [8]–[10]. In contrast, tesamorelin, a synthetic analogue of growth hormone releasing factor, is FDA approved for this indication and has only mild adverse effects on glucose homeostasis [38]–[41]. Rosiglitazone is either not available or restricted in its use in most parts of the world due to concerns about its potential to increase cardiovascular event rates [42]–[45]. Nonetheless, our study provides proof of concept of combining an insulin-sensitizing drug with growth hormone that could be the basis for future studies combining related drugs.

## Supporting Information

Appendix S1 **Sample size calculations.**
(DOCX)

Checklist S1 **CONSORT 2010 checklist of information to include when reporting a randomised trial.**
(DOC)

Protocol S1 **Study protocol.**
(PDF)

Table S1 **Baseline and Change at Week 12 in Body Weight and Body Composition Parameters by Dual Energy Absorptiometry and Stable Isotope Dilution.**
(DOC)
