# Supplementary material for: Recombinant Human Growth Hormone and Rosiglitazone for Abdominal Fat Accumulation in HIV-Infected Patients with Insulin Resistance: A Randomized, Double-Blind, Placebo-Controlled, Factorial Trial
Source: PLoS One. 2013 Apr 12;8(4):e61160. doi: 10.1371/journal.pone.0061160 (PMC3625151; doi:10.1371/journal.pone.0061160)
Supplement: Appendix S1 — Sample size calculations. (DOCX) [file pone.0061160.s001.docx]

Appendix

Sample Size Determination for the Primary Endpoint

We have used the publications of Gelato [1,1] and Lo [2] to construct the following table of mean changes from baseline to week 12 in SI units. Several assumptions were necessary to use these data: (1) The magnitude and variability of changes in S_I_ to be measured by FSIVGTT in the proposed study are assumed to be similar to those seen when insulin stimulated glucose uptake was measured by euglycemic hyperinsulinemic clamp studies by Gelato and Lo; (2) The Rd or M values (glucose uptake) reported by Gelato were divided by the targeted insulin concentration in that study of 40 uU/ml to obtain estimated M/I values (the outcome reported by Lo et al); and, (3) Since Lo’s study did not have a week 12 time point, week 4 data were used instead. After converting the data from these two studies to the same units of mg glucose/kg LBM/min/uU insulin/ml, the net changes in M/I from baseline to week 12 or week 4 were -0.31 in the Lo study of rhGH and +0.055 in the Gelato study of rosiglitazone. These were multiplied by a factor of 100 for the power calculation, as outlined in the table below:

Where x might vary from -30 to +60 (i.e. a range defined by no effect of rosiglitazone when combined with rhGH and no effect of rhGH when combined with rosiglitazone).

|  |  | Rosiglitazone | |
| --- | --- | --- | --- |
|  |  | Yes | No |
| rhGH | Yes | x | -31 |
|  | No | 55 | 0 |

We assume that with 20 subjects in each cell and a 20% premature discontinuation rate, 16 subjects in each cell will complete week 12 of the study. To calculate power for the proposed 16 subjects in each cell (total of 64 subjects), the x-values used were -30, -20, -10, 0, 10, 20, 30, 40, 50, 60. A standard deviation of 30 was assumed (calculated by pooling the standard deviations from the Lo and Gelato studies).

The following table shows the power for detecting interactions and main effects for the various values of x in the 2x2 design:

| SD=30  Correlation=0  N=16 | x-value  (rhGH x Rosi cell) | Power for rhGH main effect | Power for Rosiglitazone main effect | Power to detect an interaction between rhGH and Rosiglitazone |
| --- | --- | --- | --- | --- |
|  | -30 | 99% | 95% | 94% |
|  | -20 | 99% | 99% | 82% |
|  | -10 | 99% | 99% | 60% |
|  | 0 | 99% | 99% | 42% |
|  | 10 | 99% | 99% | 15% |
|  | 20 | 99% | 99% | 5% |
|  | 30 | 95% | 99% | 6% |
|  | 40 | 85% | 99% | 18% |
|  | 50 | 65% | 99% | 39% |
|  | 60 | 39% | 99% | 75% |

It is clear that the statistical power for detecting main effects is generally quite high. Although the statistical power for detecting interactions ranges from very low to high values, we believe that, under the assumption that the effect of rhGH is ‘stronger’ than that of rosiglitazone, x values of –20 to –10 are reasonable and yield power in the range of 60 to 82 percent.

Sample Size Determination for Secondary Endpoint of Change in VAT

We performed sample size calculations for this aim based on our preliminary data on the expected change in the levels of VAT at week 12, which was –2.5 L at a dose of rhGH of 6 mg/d [3]. The available data suggest that the dose-response curve of rhGH on VAT is relatively linear; by cross-sectional CT, subjects receiving 4 mg/d of rhGH had ~35 cm^2^ mean reduction in VAT compared with ~18 cm^2^ at a dose of 4 mg every other day in the phase II study of rhGH for visceral adiposity [4,5]. Therefore, we estimated that the change in VAT at the 3 mg/d dose proposed here would be 50% of that seen at the 6 mg/d dose, or –1.25 L. The data on changes in VAT in HIV-infected patients receiving rosiglitazone are conflicting, with two studies showing no change [6] and another showing a 21% decrease [1]. For the purpose of this calculation, we estimated a decrease of 10% (-0.5 L) in the rosiglitazone + rhGH placebo arm. We assumed that rosiglitazone would not add to the effects of rhGH in the combination arm and that there would be no change in the double-placebo arm. Using a similar approach to the insulin sensitivity aim, the basis of the power calculation for the body composition aim is summarized in the following table of changes in VAT at week 12:

|  |  | Rosiglitazone | |
| --- | --- | --- | --- |
|  |  | Yes | No |
| rhGH | Yes | -1.25 | -1.25 |
|  | No | -0.5 | 0.00 |

The following table shows the power for detecting interactions and main effects based on the above assumptions and varying values of the n with evaluable data:

| SD=0.40  Correlation=0 | N | Power for rhGH main effect | Power for Rosiglitazone main effect | Power to detect an interaction between rhGH and Rosiglitazone | |
| --- | --- | --- | --- | --- | --- |
|  | 12 | 99% | 56% | 56% |  |
|  | 14 | 99% | 63% | 63% |  |
|  | 16 | 99% | 69% | 69% |  |
|  | 20 | 99% | 78% | 78% |  |

With 16 evaluable subjects, the power for detecting the main effect of rhGH is high and somewhat lower for rosiglitazone and the interaction.

References

1. Gelato MC, Mynarcik DC, Quick JL, Steigbigel RT, Fuhrer J, et al. (2002) Improved insulin sensitivity and body fat distribution in HIV-infected patients treated with rosiglitazone: a pilot study. J Acquir Immune Defic Syndr 31: 163-170.

2. Lo JC, Mulligan K, Noor MA, Schwarz JM, Halvorsen RA, et al. (2001) The effects of recombinant human growth hormone on body composition and glucose metabolism in HIV-infected patients with fat accumulation. J Clin Endocrinol Metab 86: 3480-3487.

3. Engelson ES, Glesby MJ, Mendez D, Albu JB, Wang J, et al. (2002) Effect of recombinant human growth hormone in the treatment of visceral fat accumulation in HIV infection. J Acquir Immune Defic Syndr 30: 379-391.

4. Kotler DP, Thompson M, Grunfeld C, Testa M, Turner R, et al. (2002) Growth Hormone (Serostim) effectively reduces viceral adipose tissue (VAT) accumulation and non-HDL cholesterol. Program and Abstracts of the XIV World AIDS Conference, Barcleona, Spain, July 7-12, 2002 abstr LbOr18.

5. Kotler DP, Muurahainen N, Grunfeld C, Wanke C, Thompson M, et al. (2004) Effects of growth hormone on abnormal visceral adipose tissue accumulation and dyslipidemia in HIV-infected patients. J Acquir Immune Defic Syndr 35: 239-252.

6. Yki-Jarvinen H, Sutinen J, Silveira A, Korsheninnikova E, Fisher RM, et al. (2003) Regulation of Plasma PAI-1 Concentrations in HAART-Associated Lipodystrophy During Rosiglitazone Therapy. Arterioscler Thromb Vasc Biol 23: 688-694.
