# Supplementary material for: Recombinant Human Growth Hormone and Rosiglitazone for Abdominal Fat Accumulation in HIV-Infected Patients with Insulin Resistance: A Randomized, Double-Blind, Placebo-Controlled, Factorial Trial
Source: PLoS One. 2013 Apr 12;8(4):e61160. doi: 10.1371/journal.pone.0061160 (PMC3625151; doi:10.1371/journal.pone.0061160)
Supplement: Protocol S1 — Study protocol. (PDF) [file pone.0061160.s003.pdf]

Randomized, Double-Blind, Placebo-Controlled Study of the Safety and Efficacy of Recombinant Human Growth Hormone and/or Rosiglitazone in the Treatment of Human Immunodeficiency Virus-Associated Visceral Adiposity and Insulin Resistance

Protocol Chair: Marshall J. Glesby, MD, PhD
Cornell Clinical Trials Unit
Division of International Medicine and Infectious Diseases
Department of Medicine
Weill Medical College of Cornell University
525 E. 68th Street, Box 566
New York, NY 10021

Phone: 212-746-7134
Fax: 212-746-8852
Email: mag2005@med.cornell.edu
Pager: 888-773-4536

Participating Sites:

Columbia University College of Physicians and Surgeons (New York Presbyterian Hospital)
St. Luke's-Roosevelt Hospital Center
Weill Medical College of Cornell University (New York Presbyterian Hospital)

Version 3.0
February 10, 2005

Table of Contents

1.0 Synopsis 3

2.0 Background 4

3.0 Objectives 12

4.0 Entry Criteria 13

5.0 Study Medications 15

6.0 Study Procedures 20

7.0 Toxicity Management 27

8.0 Criteria for Permanent Discontinuation 30

9.0 Adverse Event Reporting 30

10.0 Study Monitoring 32

11.0 Data Entry and Management 31

12.0 Statistical Considerations.. ……………………… 32

APPENDIX 1: SCHEDULE OF EVENTS 41

APPENDIX 2: ORAL GLUCOSE TOLERANCE TEST METHODOLOGY 45

APPENDIX 3: ANTHROPOMETRIC MEASUREMENTS 44

APPENDIX 4: SAMPLE INFORMED CONSENT FORM 47

1.0 Synopsis

Design:
The overall experimental design is a randomized, double-blind, multicenter study evaluating the safety and efficacy of recombinant human growth hormone (rhGH), rosiglitazone, or both, compared with no active therapy (double-placebo), for the treatment of HIV-associated visceral adiposity in subjects with insulin resistance by quantitative insulin sensitivity index (QUICKI). Within this clinical trial, we will perform careful metabolic assessments on the subjects in each arm over time to test hypotheses about the effects of the specific therapeutic regimens on metabolic parameters.

The study will be conducted in the General Clinical Research Centers (GCRCs) of both campuses of the New York Presbyterian Hospital (New York Weill Cornell Medical Center and Columbia Presbyterian Medical Center) and St. Luke's-Roosevelt Hospital Center.

Subjects:
Eligible subjects will meet anthropometric criteria for probable visceral adiposity and have a QUICKI value consistent with insulin resistance. Subjects must be on stable antiretroviral therapy and must not have diabetes or other contraindications to rhGH or rosiglitazone therapy.

Regimens:
Consenting subjects will be randomized initially in a 1:1:1:1 ratio to one of the following arms:

Arm A (n = 20):	
Weeks 0-12 (double-blinded):	rhGH 3 mg s.c. qd PLUS rosiglitazone 4 mg po bid
Weeks 13-24 (open-label):	rhGH 2 mg s.c. qod PLUS rosiglitazone 4 mg po bid
Arm B (n = 20):	
Weeks 0-12 (double-blinded):	rhGH 3 mg s.c. qd PLUS rosiglitazone placebo 1 po bid
Weeks 13-24 (open-label):	rhGH 2 mg s.c. qod PLUS rosiglitazone 4 mg po bid
Arm C (n = 20):	
Weeks 0-12 (double-blinded):	rhGH placebo s.c. qd PLUS rosiglitazone 4 mg po bid
Weeks 13-24 (open-label):	rhGH 2 mg s.c. qod PLUS rosiglitazone 4 mg po bid
Arm D (n = 20):	
Weeks 0-12 (double-blinded):	rhGH placebo s.c. qd PLUS rosiglitazone placebo 1 po bid
Weeks 13-24 (open-label):	rhGH 2 mg s.c. qod PLUS rosiglitazone 4 mg po bid

Randomization will be stratified on center and on the presence or absence of impaired glucose tolerance (based on the results of the screening oral glucose tolerance test [OGTT]). Upon completion of the initial 12 week period of blinded dosing, all subjects will receive open-label low dose rhGH and rosiglitazone for 12 additional weeks as above.

Procedures:
All subjects will have OGTTs with insulin levels at screening, ~~entry~~**,** weeks 4, 12, and 24 and frequently sampled intravenous glucose tolerance tests (FSIVGTTs) at entry and week 12. Basal free fatty acid (FFA) flux, resting energy expenditure (REE), and energy intake will also be determined at entry and week 12. Subjects will undergo total body MRI scanning at SLRHC at entry, week 12, and week 24 to measure visceral and subcutaneous adipose tissue. Subjects will also have dual energy X-ray absorptiometry (DEXA) scans and sodium bromide and deuterated water dilution tests done at entry and week 12.

2.0 Background

Visceral Adiposity and Lipodystrophy in HIV-Infected Patients
Reports of metabolic derangements in HIV-infected patients, including aberrations in glucose and lipid metabolism and body fat distribution, became prevalent about 18 months after the licensure of the first protease inhibitor in the United States. Investigators and clinicians have used a number of terms to characterize patients with changes in fat distribution, the most common of which have been *lipodystrophy* or *lipodystrophy syndrome*. Affected patients may have fat wasting (lipoatrophy), central fat accumulation, or a mixed picture of fat wasting and accumulation. The fat wasting component consists of thinning of the face, buttocks, arms, and/or legs with prominence of veins due to subcutaneous fat loss, whereas the fat accumulation component consists of increased abdominal girth, breast enlargement, and/or dorsocervical and submandibular fat pad enlargement [5,35,42]. Researchers have used dual energy X-ray absorptiometry (DXA) to confirm the abnormal distribution of fat [5,6] and computed tomography and whole body magnetic resonance imaging to document the visceral nature of the abdominal fat accumulation [10]. Incidence data for these alterations in fat distribution are limited due to the cross-sectional nature of most studies, and estimates of prevalence range widely from 7% to 83% [6,54]. A major limitation of studies done to date is the lack of a consistent case definition for the syndrome or syndromes. Most studies rely on patient self-report or clinician assessment for diagnosis. Similarly, no consensus has been reached on nomenclature.

Recent preliminary data from men participating in the Fat Redistribution and Metabolic Change (FRAM) study have questioned whether fat accumulation/visceral adiposity is statistically associated with lipoatrophy in HIV-infected patients [19]. FRAM is a cross-sectional, epidemiological study aimed at deriving a case definition for “lipodystrophy” by comparing randomly selected HIV-infected patients to HIV-uninfected controls from the CARDIA study. As expected, self-reported and clinically assessed peripheral lipoatrophy was more prevalent among the HIV-infected men compared with controls. Surprisingly, central fat *loss* rather than accumulation was also more prevalent among the HIV-infected men, as confirmed by DXA and MRI studies. Although the cross-sectional nature of FRAM is a major limitation, its rigorous methodology and lack of assumptions about which phenotypic features of the syndrome(s) are truly linked are major strengths**.** Preliminary data from the Women’s Interagency Health Study and the CPCRA FIRST study have yielded similar results [60]. Importantly, the preliminary findings from these studies do not controvert the presence of visceral adiposity in some HIV-infected patients but rather question its association with lipoatrophy. Visceral adiposity may, in

fact, be an independent complication of HIV disease or its therapy or perhaps may even represent the co-existence of the metabolic syndrome (Syndrome X) that is prevalent in the general population [2,11]. Even if visceral adiposity and lipoatrophy are not part of the same syndrome, the metabolic consequences of excess visceral adiposity remain of concern in the HIV-infected population.

Multiple studies in the general population have demonstrated independent associations between visceral adipose tissue area (VAT) and various components of the metabolic syndrome after adjusting for overall adiposity. VAT is associated with insulin levels [51,67] (and inversely with insulin sensitivity [7]), glucose levels [67], systolic and diastolic blood pressure [67], and levels of triglycerides, LDL-cholesterol, HDL-cholesterol, apolipoprotein B, and apolipoprotein A-1 [13,14,51]. Whether visceral adiposity causes insulin resistance or is only another marker of insulin resistance is unclear. Nontheless, VAT may be the best correlate of the atherogenic complications seen in the metabolic syndrome, namely the dyslipidemia and insulin resistance [32]. Inflammatory markers that are predictive of cardiovascular events, such as C-reactive protein, may also be linked to visceral adiposity [33]. Taken together, the available data suggest that visceral adiposity may play a central role in the metabolic syndrome. By extension, visceral adiposity may also play a central role in the constellation of metabolic abnormalities that are prevalent in HIV-infected patients. If so, visceral adiposity is an attractive target for intervention.

Cardiovascular Risk in HIV Infection

Recent epidemiologic studies suggest that HIV-infected subjects may have an increased incidence of myocardial infarction relative to the general population. Klein et al retrospectively analyzed hospital admission data from the Kaiser Permanente database in California and reported an approximately two-fold increased incidence of admission for coronary heart disease among HIV-infected patients compared with those not known to be HIV-infected [29]. The hospitalization rate did not differ among the HIV-infected patients when stratified by protease inhibitor use. Mary-Krause et al retrospectively reviewed data from over 60 hospitals in France and reported an increased rate of myocardial infarction in HIV-infected patients on protease inhibitors for over 18 months compared with incidence data from the general population [38]. Longer duration of protease inhibitor use was associated with an increased relative risk of myocardial infarction in this study. Data from the HIV Outpatient Study also suggest that PI use is associated with an increased risk of myocardial infarction [25]. Recent preliminary results from the D:A:D cohort study, a compilation of data from 11 international cohorts with over 23,000 HIV-infected patients, found a 26% increased risk of myocardial infarction per year of exposure to combination antiretroviral therapy, adjusted for other cardiovascular risk factors [12]. In contrast, Bozzette et al reported conducted a retrospective analysis of data from the Veterans' Administration hospitals and found that the overall dramatic reduction in mortality attributable to antiretroviral therapy was not abrogated by increased rates of cardiovascular or cerebrovascular admissions or deaths [3]. Although data are not yet available on the incidence of ischemic cardiac events specifically in patients with lipodystrophy, Wilkie et al recently presented data demonstrating that 22 HIV-infected women with lipodystrophy had increased carotid intima medial thickness, a marker of subclinical atherosclerosis, compared to age, sex, and race-matched controls [65]. In contrast, in a large cross-sectional study, conventional

cardiovascular risk factors rather than diagnosis of lipodystrophy per se was associated with increased carotid intima medial thickness in HIV-infected patients [41].
HIV-infected patients with visceral adiposity/lipodystrophy have multiple phenotypic features of the metabolic syndrome that is associated with accelerated atherosclerosis. Hadigan et al compared clinical features of 71 HIV-infected patients with lipodystrophy (defined as a change in fat distribution in $\geq$ one body area) with 213 age and body mass index-matched healthy controls from the Framingham Offspring Study and compared 30 HIV-infected patients without lipodystrophy to an additional 90 matched healthy controls [23]. Compared with the Framingham controls, the lipodystrophy patients had increased waist to hip ratios and increaed fasting insulin, cholesterol, triglyceride, and 2-hour glucose and insulin levels on OGTT. Diastolic blood pressure was also higher and HDL cholesterol levels lower. In contrast, the metabolic profiles of the HIV-infected patients without lipodystrophy were similar to the Framingham controls. The investigators concluded that HIV-infected patients with lipodystrophy have metabolic abnormalities (i.e. insulin resistance, dyslipidemia, truncal adiposity, and increased diastolic blood pressure) that may predispose them to acclerated cardiovascular disease. In a similar matched analysis, the same investigators demonstrated markedly elevated levels of tissue plasminogen activator (tPA) and plasminogen activator inhibitor-1 (PAI-1), markers of impaired fibrinolysis, in patients with lipodystrophy [24]. These data suggest a potential pathogenetic link between the hyperinsulinemia/insulin resistance that is prevalent in patients with lipodystrophy and risk of cardiovascular events, as seen in the Framingham cohort [40].

Thus, the available data suggest that HIV-infected patients with visceral adiposity have a metabolic profile that may predispose to accelerated atherosclerosis. Interventions targeted at improving insulin sensitivity and reducing visceral fat have the potential to favorably modify the risk of cardiovascular disease in this population.

<u>Interventions for HIV-Associated Visceral Adiposity</u>
Data on interventions for visceral adiposity in HIV-infected patients are limited. Most studies of switching antiretrovirals, usually from a PI to a nonnucleoside reverse transcriptase inhibitor or abacavir, have demonstrated improvements in lipid profiles and insulin resistance but minimal or no impact on truncal fat [36,37]. Most of these studies have had methodologic limitations, specifically lack of randomization or subjective endpoints [18]. Limited data on exercise have demonstrated modest improvements in truncal adiposity [52,53]. There is a clear need for safe and effective treatment of visceral adiposity.

<u>Growth Hormone</u>
Recombinant human growth hormone (rhGH) is currently approved in the United States for treatment of AIDS wasting/cachexia. Because of its lipolytic effects [46], rhGH has been studied in several open-label trials as treatment for HIV-associated visceral adiposity. Torres and colleagues [61] administered rhGH to 10 patients with increased abdominal girth and/or buffalo humps at doses of 5-6 mg/d subcutaneously. Nine patients had subjective responses but 2 developed new onset or worsening of hyperglycemia. Similarly, in a prospective, open-label study, Wanke et al [64] determined the efficacy of 12 weeks of treatment with rhGH (6 mg/d) in the treatment of lipodystrophy (defined as self-reported or clinician reported fat redistribution) in 10 patients. Waist/hip ratio significantly decreased from baseline levels after treatment with

growth hormone (1.03 to 0.9; p<0.04). One patient had to discontinue therapy because of hyperglycemia. In an unpublished double-blind, placebo-controlled, crossover trial, Furrer et al. [15] showed that 6 mg/d of rhGH significantly reduced truncal fat but also was associated with significant adverse effects. In the largest published study to date, we demonstrated significant reductions in visceral adipose tissue (VAT), mild decreases in subcutaneous adipose tissue (SAT), but a high incidence of diabetes in 30 subjects with visceral adiposity treated initially with 6 mg/d of rhGH [9]. The effect of rhGH on VAT was near maximal at 12 weeks and VAT re-accumulated to near-baseline 12 weeks after cessation of a 24-week course of rhGH in this study.

Lo et al treated 8 HIV-infected patients with enlarged dorsocervical fat pads and/or abdominal obesity with rhGH 3 mg/d for 6 months [34]. Treatment was discontinued in one patient who developed diabetes within 2 weeks of starting rhGH and who was retrospectively noted to have had an oral glucose tolerance test result consistent with diabetes prior to starting rhGH. Two subjects did not complete the study for reasons unrelated to treatment and one had a 50% reduction in rhGH dose for arthralgias. The latter patient had an OGTT result consistent with diabetes at month 1 that improved at month 6 (after dose reduction). Insulin-stimulated glucose uptake, assessed by euglycemic hyperinsulinemic clamp, decreased significantly from baseline to month 1 (49.7 ± 27.5 to 25.6 ± 6.6 nmol/kg_{LBM}.min/$pmol_{INSULIN}$/L) but returned to baseline at month 6 (49.2 ± 22.6). Area under the curve glucose from the OGTTs also increased at month 1 and improved by month 6 but remained higher than baseline. As assessed by DXA, completing subjects showed a mean decrease in total fat of 4.4 kg (primarily from the trunk) and a mean increase in lean body mass of 5.4 kg. At 6 months, there was a trend for reduced total cholesterol levels and increased HDL cholesterol levels compared with baseline.

Kotler and colleagues have presented the preliminary results of a phase II, randomized, double-blinded, placebo-controlled trial of rhGH for lipodystrophy [31]. Eligible subjects met the same anthropometric criteria for visceral adiposity to be used in the proposed studies (see sec. D.2.c.) and had normal OGTTs and fasting glucose levels. They were randomized 1:1:1 to rhGH 4 mg/d (n=82), rhGH 4 mg qod (n=79), or placebo (n=78) for 12 weeks. By CT scanning at the L4-L5 level, VAT decreased significantly in the 4 mg/d arm ($p < 0.001$ from baseline) and to a lesser degree in the 4 mg qod arm ($p = .052$). Of note, VAT did not change significantly over a 12 week period in the placebo arm. Similar trends were seen in changes in trunk:limb fat ratio by DXA. Mean non-HDL cholesterol also decreased significantly in the active treatment arms (-18 mg/dL and –12 mg/dL in the qd and qod arms, respectively). At week 12, dosing was changed for the subsequent 12 weeks such that placebo recipients started rhGH 4 mg/d and those receiving rhGH 4 mg/d were re-randomized to rhGH 4 mg qod or placebo; those originally receiving 4 mg qod continued on this dose. Areas under the curve for insulin and glucose on OGTT increased at week 12 but returned to baseline at week 24 in the two arms that continued on rhGH for 24 weeks [30]. These results are consistent with data on rhGH treatment of viscerally obese HIV-uninfected men where insulin sensitivity initially worsens but then returns to or below baseline with ongoing therapy [26]. The hypothesized explanation for this biphasic change is that rhGH decreases insulin sensitivity initially but the subsequent reduction in visceral fat leads to increased insulin sensitivity.

Taken together, these data suggest that rhGH is a potentially effective treatment for visceral adiposity in HIV-infected patients and the associated dyslipidemia, but its adverse effects on insulin sensitivity may limit its usefulness in a patient population with a high underlying prevalence of insulin resistance.

B.3.b. Thiazolidinediones
There is considerable interest in the use of insulin-sensitizing agents in HIV-infected patients with abnormal fat distribution, especially those with documented insulin resistance or diabetes. The thiazolidinedione drugs are ligands for peroxisome proliferator-activated receptor-gamma (PPAR-gamma), which plays a role in the differentiation of pre-adipocytes to adipocytes. These drugs are believed to act primarily at the level of muscle to improve insulin sensitivity [50]. There are currently two thiazolidinedione drugs on the market, rosiglitazone and pioglitazone. A third drug in this class, troglitazone, was removed from the market because of cases of fatal hepatotoxicity in association with its use; this association has not been observed with either rosiglitazone or pioglitazone. Some of the available data pertinent to the proposed work, however, are on troglitazone.

Because of its activity in adipose tissue, investigators have studied the effects of troglitazone on body composition in type 2 diabetics or subjects with visceral adiposity. In one study of 30 diabetic patients treated for 6 months, VAT decreased from 118.3 ± 54.3 to 101.1 ± 50.8 cm^2 and SAT increased from 189.7 ± 93.3 to 221.6 ± 101.6 cm^2 [45]. In a smaller study, 10 diabetic men treated with dietary advice and troglitazone tended to have reductions in VAT (mean 132.8 ± 21.3 to 122.6 ± 22.1) and increases in SAT (121.1 ± 13.5 to 126.0 ± 17.4) at 6 months [27]. Similar trends were seen in a placebo-controlled study of troglitazone in subjects with visceral adiposity [48].

Troglitazone has also been studied in HIV-uninfected patients with lipodystrophy syndromes. In one report, 20 patients with various syndromes associated with lipoatrophy or lipodystrophy were treated with troglitazone for 6 months [1]. MRI scanning demonstrated increases in SAT but not VAT, and those with diabetes had improved glycemic control.

Troglitazone has been studied in 6 HIV-infected patients with abnormal fat distribution and protease inhibitor-associated diabetes mellitus and was found to improve insulin sensitivity in 4 of them [63]. Furthermore, 4 patients had decreases in VAT and 4 had increases in SAT. Yki-Järvinen et al conducted a randomized, 24-week, placebo-controlled study of rosiglitazone 8 mg/d in 30 subjects with lipodystrophy (defined as self-reported and investigator-confirmed body shape changes)[58]. Mean serum insulin levels fell from 13 ± 2 (SEM) to 9 ± 1 mU/L in the rosiglitazone arm compared with an increase from 10 ± 2 to 16 ± 6 mU/L in the placebo arm. Visceral and subcutaneous fat, assessed by MRI, however, did not change significantly in either arm. The authors concluded that rosiglitazone ameliorates insulin resistance but not the body composition changes in lipodystrophy. Of note, the baseline mean VAT and insulin levels were both considerably lower than those in the patients studied by us in a pilot study of rhGH (see Preliminary Studies) and may not be entirely representative of the patient population targeted by the proposed study.

Gelato et al reported on the effects of rosiglitazone 8 mg/d in 9 HIV-infected subjects with

insulin resistance documented by hyperinsulinemic-euglycemic clamp [16]. One subject was withdrawn due to hepatotoxicity while the remaining 6 subjects completed at least 6 weeks of treatment. As expected, insulin sensitivity increased with rosiglitazone treatment. In addition, SAT, assessed by single slice CT at the L4 level, increased by a mean of 23 ±10% and VAT decreased by a mean of 21% ± 8%. Of note, the subjects in this study were not selected based on body composition changes, though compared with HIV-uninfected controls, they did have decreased proportions of limb fat relative to total body fat. Hadigan et al recently presented results of a randomized, placebo-controlled study of rosiglitazone (initially dosed at 4 mg qd) in 28 HIV-infected subjects with hyperinsulinemia and lipoatrophy [21]. In the rosiglitazone arm, insulin sensitivity improved and SAT by abdominal CT increased by 8% while VAT was unchanged.

Individual Effects of Growth Hormone and Thiazolidinediones on Lipolysis and Energy Expenditure

The available data suggest that growth hormone adversely affects insulin sensitivity primarily by stimulating lipolysis and increasing free fatty acid (FFA) concentrations. For example, in growth hormone-deficient adults receiving rhGH replacement, co-administration of acipimox, a nicotinic acid derivative that inhibits lipolysis at the level of hormone-sensitive lipase, abrogated the effects of rhGH on insulin sensitivity [49,55]. Thiazolidinediones (TZDs) have pleiotropic effects that likely account for their favorable effects on insulin sensitivity; however, their effects on suppressing lipolysis and reducing FFA concentrations may play a major role in this regard [39,43]. Recent data provide insight into a key mechanism by which TZDs reduce circulating FFAs. TZDs induce expression of glycerol kinase in adipocytes, which stimulates glycerol incorporation into triglycerides and reduces FFA secretion from adipocytes. This takes place even during stimulation of lipolysis by a beta agonist [20]. These data provide biological plausibility for our hypothesis that rosiglitazone will reduce FFA levels even in the presence of rhGH-induced stimulation of lipolysis and support the rationale of combination therapy with rosiglitazone and rhGH.

Growth hormone increases resting energy expenditure (REE) in diverse patient populations, including patients with HIV-associated wasting [47]. In metabolic ward studies of wasted patients treated with supraphysiologic doses of rhGH (0.1 mg/kg/d), there was no significant change in energy intake after adjustment for changes in weight or lean body mass (LBM), suggesting that the rhGH-induced increase in LBM was due to redistribution of energy stores from fat. Although total energy expenditure (TEE) was not measured, the authors felt that a decrease in overall activity levels leading to constant TEE (and energy balance) was an unlikely explanation for their findings. To our knowledge, no data are available on the effects of TZDs on energy expenditure and balance. However, the gains in body weight and SAT reported with TZD use imply that these drugs drive energy balance in the positive direction. The current studies will fill a gap in our knowledge of the effects of TZDs on energy balance in HIV-infected patients with visceral adiposity, as well as the net effect of the combination of TZDs and rhGH.

Combination Therapy with Thiazolidinediones and Growth Hormone

Investigators have studied the co-administration of rhGH and troglitazone in male Wistar rats and found that troglitazone blocked the antagonistic effects of rhGH on hepatic glucose output and peripheral glucose utilization [57]. Similarly, pre-treatment of female ob/ob mice with

pioglitazone was found to prevent rhGH-induced increases in blood glucose and insulin levels, while these levels were reduced by pioglitazone treatment after initial administration of rhGH [62]. To our knowledge, no human data exist on co-administration of thiazolidinediones and rhGH, which emphasizes the importance of the proposed studies.

Significance of Proposed Studies

Visceral adiposity is a clinically important condition in a significant proportion of HIV-infected patients. The proposed studies will test the safety and efficacy of a novel approach to treating these abnormalities -- combination therapy with rhGH and rosiglitazone – and compare the metabolic effects of this intervention to that of each drug alone and no active therapy (double-placebo). The studies will provide insight into the physiologic interactions of rosiglitazone and rhGH in humans and their effects on carbohydrate and lipid metabolism, energy expenditure, and body composition. If the approach under study is successful, it may ultimately lead to a safe and effective treatment strategy for HIV-associated visceral adiposity.

Study Rationale

There is currently no established therapy for HIV-associated visceral adiposity. In studies of this patient population, rhGH administration has reduced VAT significantly but is associated with worsening insulin resistance and the development of diabetes mellitus. The lipolytic effects of rhGH may also exacerbate co-existing lipoatrophy. Rosiglitazone, an insulin-sensitizing agent, may have favorable effects on both VAT and SAT. The proposed studies will investigate the effects of co-administration of rhGH and rosiglitazone versus either drug alone on insulin sensitivity, FFA metabolism, energy balance, body composition, and markers of cardiovascular risk.

The co-administration of a thiazolidinedione with rhGH to patients with visceral adiposity *may* result in several benefits: (1) improvement in underlying insulin resistance that may be exacerbated by rhGH; (2) improvement in reduction of VAT; (3) improvement in underlying subcutaneous lipoatrophy that may be exacerbated by rhGH; and, (4) reduction in cardiovascular risk. We hypothesize that combined therapy with rhGH and rosiglitazone in HIV-infected patients with visceral adiposity and insulin resistance will improve insulin sensitivity, reduce VAT, and either increase or produce no further significant reduction in SAT. These changes, we believe, will be associated with improvement in a panel of circulating factors associated with risk for cardiovascular disease, since visceral adiposity and insulin resistance are associated with multiple markers of increased cardiovascular risk.

Choice and Doses of Study Drugs

The optimal dose of rhGH for the treatment of visceral adiposity is unknown. Although our preliminary studies suggest that 6 mg/d of rhGH was efficacious at reducing VAT, it was also associated with significant toxicities. In a recently reported phase II, placebo-controlled, dose-finding study sponsored by Serono Laboratories, a dose of 4 mg/d of rhGH had superior efficacy at reducing VAT compared with 4 mg qod and placebo [31]. To reduce the likelihood of inducing diabetes mellitus, we have chosen a lower dose of 3 mg/d of rhGH, which has demonstrated favorable effects on VAT in a small open-label study [34].

Due to limited safety data on administration of rhGH at 3 mg/d for over 12 weeks, the open-label maintenance phase of this study will use a lower dose of 2 mg qod (the equivalent of 1 mg/d). Lo and colleagues reported results of a pilot study of 1 mg/d in 5 HIV-infected men with increased truncal fat and dorsocervical fat pad enlargement [35a]. Two subjects experienced mild arthralgias, one reported non-pitting edema, and a third developed carpal tunnel syndrome that necessitated premature discontinuation of therapy despite a trial of dose reduction to 0.5 mg/d. At six months, total body and trunk fat decreased in a statistically significant fashion, and lean body mass increased. VAT was reduced by 14-54% in three subjects and increased 7-10% in two subjects; overall, there was not a statistically significant change in VAT. Insulin sensitivity, as assessed by euglycemic clamp, did not change significantly. Thus, 1 mg/d of rhGH was generally well tolerated in this small, pilot study and appeared to have efficacy reducing truncal fat and increasing lean body mass, albeit to a lesser degree than seen with higher doses of rhGH.

We have chosen to use rosiglitazone for the proposed studies rather than pioglitazone because the latter is metabolized by the 3A4 isoform of cytochrome P450 (CYP 3A4), which is also involved in the metabolism of many commonly prescribed drugs for HIV infection. In contrast, rosiglitazone is not metabolized by CYP3A4 and would not be expected to interact with antiretroviral agents. We recognize that pioglitazone may have advantages over rosiglitazone, such as the more favorable effects on lipid profiles seen in diabetics [28]. We believe, however that the potential adverse consequences of drug interactions with pioglitazone outweigh any potential metabolic benefits of its use over rosiglitazone, albeit adverse effects of pioglitazone were minimal in a pilot study in 11 HIV-infected subjects [4]. Two small studies that have used 8 mg/d of rosiglitazone in HIV-infected patients did not have major safety issues [16,58]. Therefore, we have chosen to use 8 mg/d of rosiglitazone in this study.

<u>Rationale for Specific Entry Criteria</u>

<u>Presence of visceral adiposity</u>

There are currently no generally accepted and validated diagnostic criteria for visceral adiposity. It is not feasible to perform radiologic studies such as CT or MRI at screening to identify subjects with abnormal visceral fat accumulation, nor are there established criteria for diagnosing subjects by these methods. We will rely on patient self-report and clinician screening for the initial ascertainment of subjects. Their eligibility will be determined by careful measurement of waist circumference and waist to hip ratios as specified above. These criteria have been validated in HIV-infected patients and are the same as those used in a recent phase II, placebo-controlled trial of rhGH [31].

<u>Presence of insulin resistance</u>

In this study, insulin resistance will be defined as a QUICKI value ≤ 0.33. Subjects with impaired glucose tolerance on OGTT are eligible, but those who meet criteria for diabetes mellitus are excluded. The QUICKI cut-off of 0.33 was derived empirically based on our unpublished data. While other investigators have used hyperinsulinemia (e.g. fasting insulin level > 15 μU/ml [22]) to define a patient population likely to be insulin resistant, we found that QUICKI had greater sensitivity in the 30 HIV-infected subjects with increased VAT from our prior rhGH study. Although the specificity of this QUICKI definition is not known in HIV-

infected patients with visceral adiposity, we believe that its use in combination with the anthropometric criteria for increased VAT cited above will identify subjects with insulin resistance.

Antiretroviral therapy

This study will be limited to subjects who are on stable antiretroviral regimens for at least 8 weeks prior to entry, and they will be encouraged to maintain their regimens for the duration of the study unless a change is medically indicated. We feel that these criteria are not unduly restrictive so as to limit accrual but also sufficient to minimize confounding by changes in antiretroviral regimens. Although controlled studies of subjects who are randomized to switch antiretroviral regimens as an approach to treating visceral adiposity have shown somewhat limited efficacy to date, we wish to avoid changes in metabolic parameters that may be a consequence of switching antiretrovirals. We anticipate that most subjects will be taking protease inhibitors as part of their regimens but will not mandate this. We expect that protease inhibitor use will be balanced in the 3 treatment arms as a result of randomization.

Liver enzymes

Since hepatotoxicity is a potential concern with rosiglitazone, subjects must have relatively normal levels of transaminases (< 2.5 x upper limit of normal as per the drug's prescribing information) and bilirubin to enter the study. Although the presence of chronic viral hepatitis will not be exclusionary, we will test HBV and HCV serologic status at study entry to establish a baseline in the event that hepatotoxicity occurs.

3.0 Objectives:

3.1. Primary Objectives

- To determine the individual and interacting effects of rosiglitazone and rhGH on insulin sensitivity, as assessed by frequently sampled intravenous glucose tolerance test, in HIV-infected subjects with visceral adiposity and insulin resistance.
- To determine the individual and interacting effects of rosiglitazone and rhGH on body composition assessed by whole body MRI and dual energy X-ray absorptiometry (DEXA) in HIV-infected subjects with visceral adiposity and insulin resistance.

3.2. Secondary Objectives

- To determine the safety of co-administration of rosiglitazone and rhGH in HIV-infected subjects with visceral adiposity and insulin resistance
- To explore the effects of rhGH plus rosiglitazone versus either drug alone on markers of cardiovascular risk
- To determine if the effects of rhGH plus rosiglitazone versus either drug alone on insulin sensitivity will be predicted by the physiologic interaction of these drugs on free fatty acid production as assessed by ^{13}C palmitate dilution and free fatty acid suppression during oral glucose tolerance tests

- To determine if the effects of study drugs on subcutaneous adipose tissue (SAT) will differ and be determined primarily by their effects on energy balance (energy intake minus estimated total energy expenditure (TEE))
- To quantify changes in total body water (TBW) and extracellular water (ECW) over time by study arm using sodium bromide and deuterated water dilution
- To explore whether rhGH has favorable effects on self-reported fatigue, depression symptoms, and body image
- To explore the effects of rhGH plus rosiglitazone versus either drug alone on self-reported body shape

4.0 Entry Criteria

4.1. Inclusion Criteria:

4.1.1. Men and women age ≥18 years to 65 years

4.1.2. Ability and willingness of subject to provide a signed informed consent

4.1.3. HIV-1 infection, as documented by any licensed ELISA test kit, and confirmed by Western blot at any time prior to study entry. HIV-1 culture, HIV-1 antigen, plasma HIV-1 RNA, or a second antibody test by a method other than ELISA is acceptable as an alternative confirmatory test.

4.1.4. Evidence of excess abdominal adipose deposition when measured by the methodology described in Appendix 3. Use the following cut points:

- Men: Waist circumference greater than 88.2 cm AND waist:hip ratio ≥ 0.95
- Women: Waist circumference greater than 75.3 cm AND waist:hip ratio ≥ 0.9

4.1.5 Insulin resistance, defined as QUICKI ≤ 0.33. Subjects may or may not have evidence of impaired glucose tolerance, defined as 2 hour glucose level > 140 mg/dL and < 200 mg/dL on the screening oral glucose tolerance test.

4.1.6 The following laboratory values obtained within 30 days prior to study entry (unless otherwise specified):

- AST (SGOT), ALT (SGPT) ≤ 2.5 x upper limit of normal
- lipase ≤ 1 x upper limit of normal
- fasting glucose ≤ 125 mg/dL
- two hour (120 minute) glucose < 200 mg/dL on Screening Oral Glucose Tolerance Test
- fasting triglycerides ≤ 750 mg/dL

- total bilirubin ≤ 1.5 x upper limit of normal. Elevated total bilirubin < 5 mg/dL is permitted in subjects receiving indinavir or atazanavir provided that it is predominantly indirect hyperbilirubinemia.

4.1.7 Weight > 36 kg (79.3 lbs)

4.1.8 Subjects must be taking antiretroviral medications that are approved by the FDA. The regimen must have remained stable for 8 weeks prior to study entry. Subjects must also agree not to discontinue or to change their regimen for the duration of the study except as judged medically necessary.

4.1.9 All women of reproductive potential (i.e. have not reached menopause or undergone hysterectomy, oophorectomy, or tubal ligation) who participate in sexual activity that could lead to pregnancy must agree to use a form of contraception listed below for the duration of the study:

- condoms (male or female) with a spermicidal agent
- diaphragm or cervical cap with spermicide
- intrauterine device

4.1.10 Negative serum or urine pregnancy test within 14 days prior to study entry in women of reproductive potential (as defined in 4.1.9).

4.2 Exclusion Criteria:

4.2.1 Women who are pregnant or breast-feeding.

4.2.2 Active AIDS-defining opportunistic infection or untreated or suspected serious systemic infection, or persistent fever > 101°F (38.3°C) during the 30 days prior to study entry.

4.2.3 Active malignancy, except for localized cutaneous Kaposi's sarcoma (fewer than 10 lesions, none of which are larger than 2 cm, and not on active therapy).

4.2.4 Central nervous system (CNS) mass or active CNS process associated with neurological findings

4.2.5 Acute illness that is potentially life-threatening within 30 days of study entry.

4.2.6 Any condition that, in the investigator's opinion, may interfere with protocol compliance including, but not limited to, active substance abuse and/or dementia.

4.2.7 Any prior diagnosis of one of the following conditions:

- pancreatitis
- carpal tunnel syndrome (unless resolved by surgical release)

- diabetes mellitus
- angina pectoris
- coronary artery disease
- any disorder associated with moderate to severe edema (e.g., cirrhosis, nephrotic syndrome, congestive heart failure, lymphedema)

4.2.8 Untreated hypertension (defined as systolic blood pressure > 140 mm Hg or diastolic blood pressure > 90 mm Hg) within 30 days of entry.

4.2.9 Uncontrolled hypertension (defined as systolic blood pressure > 180 mm Hg or diastolic blood pressure > 115 mm Hg) within 30 days of entry.

4.2.10 Use of any of the following medications within 12 weeks of study entry:

- Therapy for obesity including therapy with anorexigenic or fat reducing drugs
- Anti-diabetic or insulin sensitizing medications including rosiglitazone, troglitazone, pioglitazone, or metformin
- Systemic glucocorticoids
- Growth hormone or any medication for AIDS-associated wasting
- Systemic chemotherapy, interferon, or radiation therapy
- Androgenic agents such as testosterone, nandrolone (Deca-durabolin), oxandrolone (Oxandrin), etc. (Testosterone replacement therapy for hypogonadism is the exception to this exclusion and will be allowed if started > 30 days prior to Entry)
- Progestational agents, unless used for oral contraception
- Appetite stimulants such as dronabinol (Marinol), megestrol acetate (Megace), or cyproheptadaine (Periactin)
- Investigational agents used under another protocol, unless discussed and approved in advance by the study chair.

4.2.11 Use of lipid lowering agents started < 12 weeks prior to entry. (Use of such drugs is permitted if begun ≥ 12 weeks prior to entry.)

4.2.12 Allergy or hypersensitivity to growth hormone, rosiglitazone, or components of these study drugs.

4.2.13 Any contraindication to MRI scanning (e.g. cardiac pacemaker, intracranial aneurysm clips).

5.0 Study Medications

Regimens

Consenting subjects will be randomized initially in a 1:1:1:1 ratio to one of the following arms:

Arm A (n = 20):
Weeks 0-12 (double-blinded): rhGH 3 mg s.c. qd PLUS rosiglitazone 4 mg po bid
Weeks 13-24 (open-label): rhGH 2 mg s.c. qod PLUS rosiglitazone 4 mg po bid

Arm B (n = 20):
Weeks 0-12 (double-blinded): rhGH 3 mg s.c. qd PLUS rosiglitazone placebo 1 po bid
Weeks 13-24 (open-label): rhGH 2 mg s.c. qod PLUS rosiglitazone 4 mg po bid

Arm C (n = 20):
Weeks 0-12 (double-blinded): rhGH placebo s.c. qd PLUS rosiglitazone 4 mg po bid
Weeks 13-24 (open-label): rhGH 2 mg s.c. qod PLUS rosiglitazone 4 mg po bid

Arm D (n = 20):
Weeks 0-12 (double-blinded): rhGH placebo s.c. qd PLUS rosiglitazone placebo 1 po bid
Weeks 13-24 (open-label): rhGH 2 mg s.c. qod PLUS rosiglitazone 4 mg po bid

Randomization will be stratified by impaired glucose tolerance versus hyperinsulinemia without impaired glucose tolerance (based on the results of the screening oral glucose tolerance test).

5.1 Drug Distribution and Accountability

rhGH will be donated by Serono Laboratories and rosiglitazone by GlaxoSmithKline. Study drugs will be stored at the Cornell Investigational Pharmacy, and a blinded 12-week supply will be dispensed to site personnel after randomization of an individual subject.

Record the dispensing of the study drug carefully, using the appropriate Drug Accountability Forms.

Include the following in the drug accountability records:

- Confirmation of study drug's delivery to the trial site
- Study drug dispensation and use by each patient
- The return to Cornell or alternative disposition of unused study drug

Records should include dates, quantities, batch numbers, expiration dates (if applicable), and any unique code numbers assigned to the investigational product(s) and/or patients. Maintain records documenting:

- That patients were provided the doses specified by the protocol
- That all study drugs provided by Cornell were fully reconciled

Do not discard unused study drug or use it for any purposes other than the present study. Instruct patients to return both used and unused study drugs, preferably in the original packs.

Do not dispense study drug to another subject once it has been dispensed to a subject and returned unused.

5.2. Concomitant Therapy

The Investigator may give the patient any medications considered necessary for the patient's welfare, unless specified below. Specifically, blood lipid lowering agents are allowed if the agents were initiated at least 30 days prior to Entry and the patient meets all other eligibility criteria. All patients must adhere to the antiretroviral therapy requirements described in the Inclusion Criteria (section 4.1.8).

The following medications are not allowed:

- Therapy for obesity including therapy with anorexigenic or fat reducing drugs
- Anti-diabetic or insulin sensitizing medications (with the exception of rosiglitazone study drug for subjects in arm B)
- Systemic glucocorticoids
- AIDS wasting therapy or growth hormone other than study drug
- Systemic chemotherapy, interferon or radiation therapy
- Androgenic agents such as testosterone, nandrolone (Deca-durabolin), oxandrolone (Oxandrin), etc. (Testosterone replacement therapy for hypogonadism is the exception to this exclusion and will be allowed if started > 30 days prior to Entry)
- Progestational agents, unless used for oral contraception
- Appetite stimulants such as dronabinol (Marinol), megestrol acetate (Megace), or cyproheptadaine (Periactin)
- Investigational agents used under another protocol, unless discussed and approved in advance by the study chair.

Report the administration of all concomitant drugs in the appropriate section of the CRF, providing dosage information, dates of administration and reasons for use. Take special care in questioning patients about self-administered medications, including over-the-counter medications and health food supplements.

Additionally, record any diagnostic, therapeutic or surgical procedure performed during the study period in the concomitant procedure section of the CRF, including the date, indication and description of the procedure(s) and outcome. Liposuction or other elective plastic surgery will not be allowed during the study.

5.3. rhGH Presentation, Preparation, Storage and Labeling

rhGH (Serostim) and matching placebo will be supplied in glass vials containing **6** mg rhGH as a sterile, non-pyrogenic white lyophilized powder. Open label rhGH will be supplied in vial sizes that will be determined at a later date. Please refer to the study manual for detailed instructions

on rhGH formulation and administration.for the blinded and open-label phases of the study. The formulation contains human recombinant growth hormone (somatropin), with sucrose and o-phosphoric acid as excipients. The pH is adjusted with sodium hydroxide or o-phosphoric acid. Upon reconstitution with 1.0 mL of supplied diluent (bacteriostatic water for injection), the solution should be clear with a pH of 6.5 to 8.5. The mixture may be gently swirled to aid dissolution. Vigorous agitation or shaking of the reconstituted vials should be avoided.

Labeling and packaging will be prepared to meet FDA requirements.

Prior to dispensing, store all study drug at room temperature, i.e., between 15° and 30° C (59° and 86°F), and in a secure location. Do not freeze the vials. If for any reason, drug is reconstituted at the site, refrigerate the reconstituted vial at temperatures between 2° and 8° C (36° and 48°F) and use it within 72 hours. Report any deviations from the recommended storage conditions to the protocol chair immediately. Interrupt use of the study drug until the protocol chair has authorized its continued use.

Only the Investigator, a member of the staff specifically authorized by the Investigator, or a pharmacist may dispense study drug.

Fax the randomization forms to the Cornell Investigational Pharmacy (centralized randomization center) to determine the treatment assignment [Fax: (212) 746-0789; person: (212) 746-0743].

5.4. rhGH Study Drug Dosing and Administration

Subjects will be treated with blinded rhGH/placebo for a total of 12 weeks. Fix dosages at 0.50 mL (3 mg rhGH) per day unless dose reduction is indicated as per the toxicity management section of the protocol.

When dispensing study drug, instruct the patient as follows:

- Store unreconstituted study drug in a secure place at room temperature, i.e., between 15° and 30° C (59° and 86° F).
- Reconstitute the vial prior to dosing, using 1mL of the diluent provided in the treatment pack. If the reconstituted drug is not used immediately, refrigerate the vial at temperatures between 2° and 8° C (36° and 48°F) and use the contents within 72 hours.
- Dose in the evenings, preferably prior to bedtime, in order to mimic the natural secretory pattern of pituitary growth hormone.
- Administer the injections subcutaneously, rotating between sites on the arms, legs, buttocks and abdomen.
- Dispose of all needles, syringes, alcohol preps, gloves and gauze used during the administration of the study drug in a puncture proof container. (The site should instruct the patient and caregiver in site procedures and policy for disposing of the container. Also instruct the caregiver in the procedures to follow in the event of a needle stick.)
- Return all used study drug vials to the site at the time of each visit.
- Return any remaining used and unused study drug vials at the end of the study.

5.5. Rosiglitazone

Rosiglitazone (Avandia) 4 mg tablets and matching placebo will be supplied by the Cornell Investigational Pharmacy. Prior to dispensing, store all study drug at room temperature, i.e., between 15° and 30° C (59° and 86° F), and in a secure location. Return any remaining used and unused rosiglitazone study drug in bottles at the end of the study.

5.7 Dispensing Study Drugs

Study drugs will be dispensed according to the schedule in Table 1 below. Pills and/or empty vials should be counted at each visit that drug is dispensed and information recorded on the drug accountability forms.

Table 1: Schedule for Dispensing Rosiglitazone (rosi) and Recombinant Human Growth Hormone (rhGH)

Study Week	Action*
Entry	Dispense rosi/placebo and rhGH/placebo
Week 2	Dispense rhGH/placebo
Week 4	Dispense rosi/placebo and rhGH
Week 8	Dispense rosi/placebo
Week 12	Dispense open-label rhGH and rosi
Week 16	Dispense open-label rhGH and rosi
Week 20	Dispense open-label rhGH and rosi

*Subject to change based on drug packaging

6.0 Study Procedures

6.1 Informed Consent

Prior to implementation of this protocol, sites must have the protocol and informed consent approved by their local institutional review board (IRB).

Once a candidate for study entry has been identified, details will be carefully discussed with the subject. The subject will be asked to read and sign the informed consent that was approved by the IRB. Written consent must be obtained prior to conducting any screening tests that are not being performed routinely for clinical management. The Informed Consent Form must be signed and personally dated by both the subject and the Investigator/Sub-Investigator. Provide a copy of the signed form to the subject and retain the original with the source documents.

6.2 Screening Visit

The Screening visit should be conducted within 30 days prior to the Entry visit (Day 1). Assign a sequential (by site) number to each patient at this time. This number will be retained throughout the patient's participation in the study. Perform the following study evaluations during this visit:

- Disease and Therapy History, including
    - mode of infection and HIV risk group (if known)
    - date of first diagnosis of HIV infection
    - date and nature of first diagnosis of all AIDS- related conditions, if any
    - all antiretroviral medications given in the year before study entry (day 1), noting dates of initiation, discontinuation and change in dose.

- Demographic Data, including
  - gender
  - date of birth
  - race/ethnicity subgroup
- Medical and Surgical History, including family history of diabetes in first degree relative.
- Current Medical Conditions, including currently active
  - disease processes
  - symptoms
- General Physical Examination including
  - body weight
  - vital signs
- Anthropometric Measurements (Appendix 3), including:
  - waist circumference
  - hip circumference
- Routine Fasting Laboratory Panels including
  - hematology
  - routine chemistry
- Fasting insulin level (after minimum 8 hour overnight fast)
- Serum or urine pregnancy test, for women of reproductive potential (see section 4.1.9 for definition)
- Oral Glucose Tolerance Test and Insulin Levels (Appendix 2). Serum and plasma **(from 5 timepoints)** will be frozen and stored locally and shipped to the Cornell GCRC Core Laboratory upon completion of the study. Draw an extra sample at the 120 minute timepoint for glucose analysis by the local laboratory. The 120 minute value done in real time by the site laboratory will be used for a final determination of eligibility (section 4.1.6).
- Plasma storage for future assays
- Review instructions for and give subject 3-day food diary to turn in at Entry visit
- Eligibility Criteria. Review the results of all Screening Evaluations prior to any final decision regarding eligibility.

6.3 Pre-Entry Visit

The pre-entry visit may be conducted any time between the Screening and Entry visits. Note: For women of child-bearing potential, a pregnancy test must be done within 7 days prior to the DEXA scan at the Entry visit. Ideally, the Pre-Entry visit should be scheduled within 7 days of Entry for these women so that the pregnancy test can be done. (If this is not possible, a stat pregnancy test can be done at SLRHC on day 1)

- **Teach subject how to inject rhGH/placebo**
- **Distribute and instruct subject in food diary (if not done at Screening visit)**
- **Instruct subject to maintain a stable diet and exercise routine (if applicable) and to abstain from strenuous activity the evening prior to the Day 1 visit and from alcohol 3 days prior to the Day 1 visit.**

- **Women of child-bearing potential: Check urine or serum pregnancy test (within 7 days of Entry).**

6.4 Entry (Days 1-2) Visit

Randomize eligible patients within 24 hours prior to the Entry Visit. Perform the following procedures and evaluations at this visit: Obtain all laboratory samples in the fasting state.

- Whole body MRI scan, DEXA scan, TBW/ECW, indirect calorimetry (resting energy expenditure) at SLRHC body composition unit on day 1
- Admission to local inpatient GCRC after completion of study procedures at SLRHC
- FSIVGTT on day 2
- FFA flux on day 2
- Physical activity structured interview (day 2)
- Current Medical Conditions (Record medical events as Current Conditions until study drug administration. After the first dose of study drug, record events as Adverse Events.)
- Concomitant Medications
- Concomitant Procedures
- General Physical Examination, including
  - body weight
  - vital signs
- Routine Fasting Laboratory Panels, including:
  - hematology
  - routine chemistry
- Serum for future determination of total testosterone and TSH
- HIV viral load, CD4
- Fasting lipid panel
- Plasma storage for future assays
- Collect completed food diary
- Lipodystrophy questionnaire
- Mental health questionnaires*

* Subjects will be given 3 standardized questionnaires about fatigue (Chalder Fatigue Scale), depression and anxiety (Brief Patient Health Questionnaire), and body image confidence (Social Physique Anxiety Scale). If any subject expresses concerns about depression, they will be offered outpatient assessment and follow-up at their site. If any subject expresses suicidal ideation on the one question assessing this symptom, they should be taken to the Emergency Room of the site for emergency psychiatric assessment. The questionnaires are brief, and are multiple choice or yes/no answers akin to symptom checklists.
They are quantitative and not qualitative and have not been shown to cause psychological distress. The Depression Questionnaire was developed for patients in Primary Medical Care settings by Spitzer et al at Columbia University and is a well validated instrument.

6.5 Study Week 2

Perform the following procedures and evaluations during the Week 2 Visit. Obtain laboratory samples in the fasting state:

- Adverse Events
- Concomitant Medications
- Concomitant Procedures
- Targeted Physical Examination, including
  - body weight
  - vital signs
- Routine Laboratory Panels, including
  - hematology
  - routine chemistry

6.6 Study Week 4

Perform these procedures and evaluations listed below during the Week 4 Visit. Obtain all laboratory samples in the fasting state:

- Adverse Events
- Concomitant Medications
- Concomitant Procedures
- Targeted Physical Examination, including
  - body weight
  - vital signs
- Routine Laboratory Panels, including
  - hematology
  - routine chemistry
- Fasting lipid panel
- Oral Glucose Tolerance Test and Insulin Levels (Appendix 2). **Collect specimens at time 0 and 120 minutes only.** Serum and plasma will be frozen and stored locally and shipped to the Cornell GCRC Core Laboratory upon completion of the study. Draw an extra sample at the 120 minute timepoint for glucose analysis by the local laboratory.
- Plasma storage for future assays
- Lipodystrophy questionnaire
- Mental health questionnaires

6.7 Study Week 6

Perform these procedures and evaluations listed below during the Week 6 Visit. Obtain all laboratory samples in the fasting state:

- Adverse Events
- Concomitant Medications
- Concomitant Procedures
- General Physical Examination, including
    - body weight
    - vital signs
- Routine Laboratory Panels, including
    - hematology
    - routine chemistry

6.8 Study Week 8

Perform these procedures and evaluations listed below during the Week 8 Visit. Obtain all laboratory samples in the fasting state:

- Adverse Events
- Concomitant Medications
- Concomitant Procedures
- General Physical Examination, including
    - body weight
    - vital signs
- Routine Fasting Laboratory Panels, including:
    - hematology
    - routine chemistry
- Fasting lipid panel
- Plasma storage for future assays
- Dispense food diary for collection at week 12

6.9 **Study Week 12**

Perform these procedures and evaluations listed below during the Week 12 Visit. Obtain all laboratory samples in the fasting state:

- Whole body MRI scan, DEXA scan, TBW/ECW, ~~OGTT~~, indirect calorimetry (resting energy expenditure) at SLRHC body composition unit on day 1
- Admission to local inpatient GCRC after completion of study procedures at SLRHC
- FSIVGTT on day 2
- FFA flux on day 2
- Physical activity structured interview (day 2)
- Collect food diary
- Adverse Events
- Concomitant Medications

- Concomitant Procedures
- General Physical Examination including
    - body weight
    - vital signs
- HIV viral load, CD4
- Routine Fasting Laboratory Panels, including:
    - hematology
    - routine chemistry
- Plasma storage for future assays
- Lipodystrophy questionnaire
- Mental health questionnaires

Oral Glucose Tolerance Test:
The Week 12 OGTT should be done on a separate day within 1 week prior to the SLHRC/local GCRC visits

- Oral Glucose Tolerance Test and Insulin Levels (Appendix 2). Serum and plasma **(from 5 timepoints)** will be frozen and stored locally and shipped to the Cornell GCRC Core Laboratory upon completion of the study. Draw an extra sample at the 120 minute timepoint for glucose analysis by the local laboratory.

6.10 Study Week 14

Perform these procedures and evaluations listed below during the Week 14 Visit. Obtain all laboratory samples in the fasting state:

- Adverse Events
- Concomitant Medications
- Concomitant Procedures
- General Physical Examination, including
    - body weight
    - vital signs
- Routine Fasting Laboratory Panels, including:
    - hematology
    - routine chemistry

6.11 Study Week 18

Perform these procedures and evaluations listed below during the Week 18 Visit. Obtain all laboratory samples in the fasting state:

- Adverse Events
- General Physical Examination, including
    - body weight
    - vital signs

- Routine Fasting Laboratory Panels, including:
  - hematology
  - routine chemistry

6.12 Study Week 24

Perform these procedures and evaluations listed below during the Week 24 Visit. Obtain all laboratory samples in the fasting state. **The Week 24 visit may be conducted over 2 days if needed.**

- Whole body MRI scan
- Adverse Events
- Concomitant Medications
- Concomitant Procedures
- General Physical Examination including
  - body weight
  - vital signs
- HIV viral load, CD4
- Routine Fasting Laboratory Panels, including:
  - hematology
  - routine chemistry
- Oral Glucose Tolerance Test and Insulin Levels (Appendix 2). Serum and plasma **(from 5 timepoints)** will be frozen and stored locally and shipped to the Cornell GCRC Core Laboratory upon completion of the study. Draw an extra sample at the 120 minute timepoint for glucose analysis by the local laboratory.

6.13 Premature Discontinuation

6.13.1 Prior to Week 4

Subjects prematurely discontinuing therapy or study participation prior to Week 4 should have the week 12 evaluations performed at the earliest possible date, with the exception of body composition assessments (MRI, DEXA, TBW/ECW).

6.13.2 After Week 4

Subjects prematurely discontinuing therapy or study participation after Week 4 should have the week 12 evaluations performed at the earliest possible date, including all body composition assessments (MRI, DEXA, TBW/ECW).

6.13.2.1 Between Weeks 12 and 16

Subjects who are on open-label study drugs who prematurely discontinue therapy or study participation prior to Week 16 should have the week 24 evaluations performed at the earliest possible date, with the exception of the MRI scan.

6.13.2.2 After Week 16

Subjects prematurely discontinuing therapy or study participation after Week 16 should have the week 24 evaluations performed at the earliest possible date, including the MRI scan.

7.0 Toxicity Management

7.1 General Issues

The main adverse effect of rhGH and rosiglitazone for which there is potential overlap is edema.

There is potential for hepatotoxicity with rosiglitazone. Although not initially recognized in clinical trials, there have been post-marketing reports of severe hepatotoxicity in association with rosiglitazone. Of note, a related drug, troglitazone, was taken off of the market because of severe hepatotoxicity in some patients resulting in death or the need for liver transplantation. Because of this, the management of liver enzyme elevation in this protocol will be conservative.

Specific clinical scenarios may necessitate dose reduction of rhGH/placebo as detailed in the following sections. Table 2 below outlines the dose reduction of rhGH/placebo.

Table 2: Dose Reduction of rhGH/Placebo

	**Dose of rhGH**
Full dose	0.50 ml (3 mg) per day
50% reduced dose	0.25 ml (1.5 mg) per day

7.2 ALT elevation

7.2.1 If a new medication with potential hepatotoxicity was started prior to the ALT elevation and the investigator believes that it may be responsible, rosiglitazone/placebo may be continued at the investigator's discretion and the protocol chair should be contacted to discuss specific management.

7.2.2 Subjects who have confirmed ALT elevation > 3 times the upper limit of normal (ULN) will permanently discontinue rosiglitazone/placebo. rhGH may be continued until the end of the study.

7.2.3 Subjects who have confirmed ALT elevation between 2.5 and 3 x ULN OR > 2 x baseline and > ULN will hold rosiglitazone/placebo and continue rhGH/placebo. The ALT should be repeated in 1 week.

If the repeat value is < 2.5 x ULN, rosiglitazone/placebo may be restarted with close monitoring.

If the repeat value is > 3 times the ULN, then rosiglitazone/placebo will be permanently discontinued; rhGH may be continued until the end of the study.

If the repeat value remains between 2.5 and 3 x ULN OR > 2 x baseline and > ULN, rosiglitazone/placebo may be restarted at the investigator's discretion with close monitoring.

7.3 **Hyperglycemia**

7.3.1 For severe hyperglycemia (symptomatic, fasting blood glucose ≥ 140 mg/dl, or 2-hour glucose on OGTT > 240 mg/dL), interrupt rhGH/placebo therapy. If the fasting blood glucose returns to < 126 mg/dL within seven days or less, resume daily dosing of rhGH/placebo at 50% of the pre-toxicity dose (Table 2). If the toxicity does not resolve within seven days, or if the severe toxicity recurs at the reduced dose of rhGH/placebo, the subject should permanently discontinue rhGH/placebo; subjects may continue rosiglitazone/placebo until the end of the study.

7.3.2 For moderate hyperglycemia (fasting blood glucose > 126 mg/dl and < 140 mg/dL or 2-hour glucose on OGTT > 200 mg/dL and < 240 mg/dL), reduce rhGH dose by 50% as per Table 1. Repeat a fasting blood glucose in 1 week.

- If the fasting glucose is still > 126 mg/dL then permanently discontinue rhGH/rosiglitazone.
- If the fasting glucose done 1 week after rhGH dose reduction is ≤ 126, then continue the reduced dose of rhGH.
- As an alternative to the above management, investigators may, at their discretion, prescribe oral antidiabetic medications that do not affect insulin sensitivity (e.g. oral hypoglycemics [sulfonylureas], acarbose).

7.4 Edema

Subjects with peripheral edema deemed by the investigator to be clinically significant should be managed in the following stepwise fashion. Management may be individualized at the discretion of the investigator.

- Counseled about reduced sodium in diet
- Prescribed a low dose of a diuretic
- Undergo dose reduction of rhGH to 50% of baseline dose
- Undergo dose reduction of rosiglitazone to 4 mg q day

7.5 Criteria for rhGH/Placebo Dose Reduction or Discontinuation

7.5.1 Severe Toxicities

7.5.1.1 Interrupt treatment with rhGH for the following severe toxicities:

- marked hypertension (symptomatic, or ≥ 200/110 mm Hg)
- serum triglycerides >1,800 mg/dl
- severe paresthesias

7.5.1.2 If the toxicity resolves within seven days or less, resume daily dosing of rhGH/placebo at 50% of the pre-toxicity dose (Table 2). If the toxicity does not resolve within seven days, or if the severe toxicity recurs at the reduced dose, the subject should permanently discontinue rhGH/placebo. Rosiglitazone/placebo may be continued until the end of the study.

7.5.1.3 Study subjects experiencing any of the following will have **all** study drugs discontinued immediately and will not be allowed to resume treatment:

- congestive heart failure
- pseudotumor cerebri (i.e., a syndrome of intracranial hypertension with papilledema, visual changes, headache, nausea and/or vomiting with no other evidence of an intracranial mass lesion or localizing neurological signs)
- a new diagnosis of cancer
- progression of an existing neoplasm, including progression of existing Kaposi's sarcoma lesions (>50% lesion growth) or the appearance of any new lesions
- severe systemic allergic manifestations (e.g., bronchospasm, laryngospasm, desquamation) thought to be related to rhGH administration
- pancreatitis

7.5.2 Moderate Toxicity

7.5.2.1 Reduce the daily dose of rhGH/placebo by 50% (Table 2) for the following moderate toxicities:

- Asymptomatic hypertension (between 140/90 and 200/110 mm Hg)
- Intolerable tissue turgor, moderate systemic allergic reaction (e.g., pruritus, erythema)
- Intolerable arthralgias not responsive to anti-inflammatory therapy
- Serum triglycerides between 1,200 mg/dl and 1,800 mg/dl
- Carpal tunnel syndrome

7.5.2.2 At the investigator’s discretion, increase the daily dose of rhGH/placebo from 50% to 100% of the initial dose if all symptoms have resolved, but return to 50% of that dose if symptoms recur. If the toxicity continues for more than 14 days at the reduced dose of rhGH/placebo, withhold treatment completely until the problem improves. If the toxicity is still unresolved following seven days without study drug dosing or after a second seven-day period of treatment at 50% of the initial dose, the subject should permanently discontinue study drugs.

7.6 Follow-Up of Adverse Events

Subjects who discontinue study drugs due to adverse events or who experience grade 3 or 4 toxicities should be followed until the resolution of the event or return to a grade 2 or lower toxicity.

8.0 Criteria for Permanent Discontinuation

Subjects must be permanently discontinued from the study if any of the following occur:

- Toxicity mandating discontinuation as detailed in section 7.
- Violation of concomitant therapy restrictions (section 5.3), including discontinuation of antiretroviral therapy
- Life-threatening illness
- New or recurrent malignancy
- Pregnancy
- Noncompliance with therapy or protocol procedures as determined by the investigator
- Study termination for administrative or safety reasons

9.0 Adverse Event Reporting

9.1 Definition

An adverse event (AE) is defined as any untoward medical occurrence in a subject that does not necessarily have a causal relationship to administration of study drug(s). An AE can therefore be any unfavorable or unintended sign (including abnormal laboratory findings), symptom, or disease temporally associated with the use of study drug(s), whether or not related to the drug(s).

9.2 Reporting

Report all AE's as defined above encountered during the study in the appropriate section of the CRF. The data must include the duration of the AE (onset/resolution dates), the severity, the relationship to study drug(s) (possible, probably, unlikely, as defined below) and any concomitant treatment dispensed or other action taken.

**Probable**	A clinical event including laboratory test abnormality with a reasonable time sequence to administration of the drug unlikely to be attributed to concurrent disease or other drugs or chemicals and which follows a clinically reasonable response on withdrawal (dechallenge). Rechallenge information is not required to fulfil this definition.

Possible A clinical event including laboratory test abnormality with a reasonable time sequence to administration of the drug but which could also be explained by concurrent disease or other drugs or chemicals. Information on drug withdrawal may be lacking or unclear.

Unlikely A clinical event including laboratory test abnormality with temporal relationship to drug administration which makes a causal relationship improbable and in which other drugs, chemicals or underlying disease provide plausible explanations.

Adverse events should be graded according to the DAIDS toxicity table (see study manual). If the event is not listed in the table, then evaluate the severity using the following four grades of severity:

Mild Subject is aware of the event or symptom but it is easily tolerated.

Moderate Subject experiences sufficient discomfort to interfere with or reduce their usual level of activity.

Severe Significant impairment of functioning; subject is unable to carry out usual activities.

Life-threatening Subject’s life was at risk from the adverse event.

9.3 Serious Adverse Events

A serious adverse event (SAE) is defined as any untoward medical occurrence that:

- Results in death
- Is life-threatening (i.e., the patient was at risk of death at the time of the event. It does not refer to an event that, hypothetically, might have caused death if it were more severe)
- Requires inpatient hospitalization or prolongation of existing hospitalization
- Results in persistent or significant disability/incapacity
- Is a congenital anomaly/birth defect
- Is another medically important condition. A medically important condition is defined as any condition that may not be immediately life threatening or result in death or hospitalization, but is clearly of major clinical significance. It may jeopardize the patient, or may require intervention to prevent one of the other serious outcomes. Examples of such events are intensive treatment in an emergency room or at home for allergic bronchospasm, blood dyscrasias or

convulsions that do not result in inpatient hospitalizations, or development of drug dependency or drug abuse.

If an SAE as defined above occurs during study treatment or within 30 days of permanent discontinuation of study drug(s), inform the protocol chair within 24 hours of learning of the event. An SAE form must also be submitted electronically or faxed to the protocol chair (212-746-8852; attention Dr. Marshall Glesby) within 24 hours of learning of the event. Follow-up information must be provided by fax or electronic submission as soon as it is available.

In addition, promptly notify the IRB and GCRC that approved the study at the local site.

10.0 Study Monitoring

There is no formal study monitoring planned. However, the protocol chair reserves the right to undertake monitoring of study documents at each site by himself or a designee.

11.0 Data Entry and Management

The Informatics Core of the Cornell GCRC will design and maintain a secure, web-based data entry system that each site will use to enter data. The system is housed on the GCRC web server (which is backed up nightly), and typically utilizes Microsoft Access as the database back-end and a combination of HTML, Javascript and ColdFusion for the web-based front-end. Access to the database is secured by both encrypted transmission (SSL) and password protection. All databases are required to be HIPAA-compliant. Where needed, methodologies exist for FDA CFR 11 compliance, including the maintenance of an audit trail for all data entry and modification. The data manager will be responsible for cleaning the dataset and querying the sites about discrepancies or missing data. Statistical analysis will be done by the Cornell GCRC statistician.

12.0 Statistical Considerations

This is a randomized, double-blind pilot study. Subjects with HIV-associated visceral adiposity will be randomized initially in a 1:1:1:1 ratio into the three arms listed below:

A) rhGH + rosiglitazone
B) rhGH + rosiglitazone placebo
C) rhGH placebo + rosiglitazone
D) rhGH placebo + rosiglitazone placebo

To ensure a similar distribution among study arms of impaired glucose tolerance (IGT), the randomization will be stratified on the presence or absence of IGT. Randomization will also be stratified by study center as is routine in multicenter clinical trials.

The primary endpoint variable for this study is the following:

1. Change in insulin sensitivity index (S_I) assessed by the FSIVGTT from baseline to week 12.

Secondary endpoint variables that will be evaluated are listed below.

1. Area under the curve (AUC) of glucose and insulin at baseline, weeks 4 and 12.
2. Percent suppression of FFAs during OGTT at baseline, weeks 4 and 12.
3. FFA flux at baseline and week 12 as measured by rate of appearance of ^{13}C-palmitate.
4. VAT and SAT at baseline and weeks 12 (and week 24 in Arm D) measured by whole body MRI scans.
5. Total body and regional fat at baseline and week 12 as measured by DXA.
6. Total intra-abdominal adipose tissue (IAAT), omental and mesenteric adipose tissue (OMAT), retroperitoneal adipose tissue (RPAT), and intramuscular adipose tissue (IMAT) at baseline and week 12 as measured by MRI scans.
7. TBW and ECW at baseline and week 12 as measured by sodium bromide and deuterated water dilution.
8. REE at baseline and week 12 as measured by indirect calorimetry.
9. Total energy balance at baseline and week 12 as calculated by energy intake (estimated by 3-day food diary records) minus (REE + estimated calories expended on physical activity)
10. Fasting lipoprotein profiles, apoB, apoA-1, hsCRP, fibrinogen, homocysteine, IL-6, tPA and PAI-1 at baseline and weeks 4 and 12.

Statistical Methods

For the primary analyses of specific aims 1-3, two-way analysis of variance (ANOVA) will be carried out to determine if the changes from baseline to week 12 in the covariates of interest differ across the three treatment arms. Initially, an F-test will be done to test for interaction. If no interaction is present, then main effects of rosiglitazone and rhGH will be assessed; if a significant interaction is present, then main effects will not be reported. For some covariates, data will be available at additional timepoints and analyzed in a similar fashion. Covariates of interest include, but are not limited to, S_I and FFA ; VAT, SAT, and energy balance; fasting lipoproteins, apolipoproteins A-1 and B, C-reactive protein (hsCRP), circulating IL-6, homocysteine, fibrinogen, tissue plasminogen activator (tPA) and plasminogen activator inhibitor-1 (PAI-1). More specifically, the data analyzed will be the changes in these variables from baseline to week 12. If the usual assumptions for ANOVA are not met (i.e. normality and homoscedasticity), then an appropriate data transformation (including a rank transformation) will be considered.

Potential confounding variables that might affect outcomes will be explored and adjusted for in the ANOVA. Since this is a study aimed at examining the biologic effects of the 3 therapies under study, the primary data analysis will use observed data only.

Safety data:

Incidence rates of adverse events and clinically significant changes in laboratory parameters will be tabulated using standard descriptive statistics.

Sample size considerations:

Sample Size Determination for the Primary Endpoint

It should first be pointed out that the specification of effect sizes for the primary outcome variable is difficult due to very limited published data. We have used the publications of Gelato [16] and Lo [34] to construct the following table of mean changes from baseline to week 12 in SI units. Several assumptions were necessary to use these data: (1) The magnitude and variability of changes in S_I to be measured by FSIVGTT in the proposed study are assumed to be similar to those seen when insulin stimulated glucose uptake was measured by euglycemic hyperinsulinemic clamp studies by Gelato and Lo; (2) The Rd or M values (glucose uptake) reported by Gelato were divided by the targeted insulin concentration in that study of 40 uU/ml to obtain estimated M/I values (the outcome reported by Lo et al); and, (3) Since Lo's study did not have a week 12 time point, week 4 data were used instead. After converting the data from these two studies to the same units of mg glucose/kg LBM/min/uU insulin/ml, the net changes in M/I from baseline to week 12 or week 4 were -0.31 in the Lo study of rhGH and +0.055 in the Gelato study of rosiglitazone. These were multiplied by a factor of 100 for the power calculation, as outlined in the table below:

		Rosiglitazone	
		Yes	No
rhGH	Yes	x	-31
	No	55	0

where x might vary from -30 to +60 (i.e. a range defined by no effect of rosiglitazone when combined with rhGH and no effect of rhGH when combined with rosiglitazone).

We assume that with 20 subjects in each cell and a 20% premature discontinuation rate, 16 subjects in each cell will complete week 12 of the study. To calculate power for the proposed 16 subjects in each cell (total of 64 subjects), the x-values used were -30, -20, -10, 0, 10, 20, 30, 40, 50, 60. A standard deviation of 30 was assumed (calculated by pooling the standard deviations from the Lo and Gelato studies).

The following table shows the power for detecting interactions and main effects for the various values of x in the 2x2 design:

	x-value (rhGH x Rosi cell)	Power for rhGH main effect	Power for Rosiglitazone main effect	Power to detect an interaction between rhGH and Rosiglitazone
SD=30<br>Corr=0<br>N=16	-30	99%	95%	94%
	-20	99%	99%	82%
	-10	99%	99%	60%
	0	99%	99%	42%
	10	99%	99%	15%
	20	99%	99%	5%
	30	95%	99%	6%
	40	85%	99%	18%
	50	65%	99%	39%
	60	39%	99%	75%

It is clear that the statistical power for detecting main effects is generally quite high. Although the statistical power for detecting interactions ranges from very low to high values, we believe

that, under the assumption that the effect of rhGH is ‘stronger’ than that of rosiglitazone, x values of –20 to –10 are reasonable and yield power in the range of 60 to 82 percent.

Sample Size Determination for Body Composition Endpoint

We performed sample size calculations for this aim based on our preliminary data on the expected change in the levels of VAT at week 12, which was –2.5 L at a dose of rhGH of 6 mg/d. The available data suggest that the dose-response curve of rhGH on VAT is relatively linear; by cross-sectional CT, subjects receiving 4 mg qd of rhGH had ~35 cm^2 mean reduction in VAT compared with ~18 cm^2 at a dose of 4 mg qod in the phase II study of rhGH for visceral adiposity [31]. Therefore, we estimated that the change in VAT at the 3 mg/d dose proposed here would be 50% of that seen at the 6 mg/d dose, or –1.25 L. The data on changes in VAT in HIV-infected patients receiving rosiglitazone are conflicting, with two studies showing no change [66] and another showing a 21% decrease [16]. For the purpose of this calculation, we estimated a decrease of 10% (-0.5 L) in the rosiglitazone + rhGH placebo arm. We assumed that rosiglitazone would not add to the effects of rhGH in the combination arm (Arm A) and that there would be no change in the double-placebo arm (Arm D). Using a similar approach to the insulin sensitivity aim, the basis of the power calculation for the body composition aim is summarized in the following table of changes in VAT at week 12:

		Rosiglitazone	
		Yes	No
rhGH	Yes	-1.25	-1.25
	No	-0.5	0.00

The following table shows the power for detecting interactions and main effects based on the above assumptions and varying values of the n with evaluable data:

SD=0.40 Corr=0	N	Power for rhGH main effect	Power for Rosiglitazone main effect	Power to detect an interaction between rhGH and Rosiglitazone
	12	99%	56%	56%
	14	99%	63%	63%
	16	99%	69%	69%
	20	99%	78%	78%

With 16 evaluable subjects, the power for detecting the main effect of rhGH is high and somewhat lower for rosiglitazone and the interaction.

Sample Size Determination for Markers of Cardiovascular Risk

No data are available to enable us to estimate the expected mean changes in markers of cardiovascular risk over time in the treatment arms of this pilot study. For simplicity, we have ignored the 2 x 2 factorial design and use a one-way ANOVA for the calculation here. If we

assume that data will be available on an average of 16 subjects in each of the 4 study arms due to premature discontinuations, then the one way ANOVA will have 80% power for detecting an effect size of 0.2, which is considered a “small” effect size [8]. In other words, there will be good power to detect small differences among the groups.

REFERENCES

1. Arioglu E, Duncan-Morin J, Sebring N et al. Efficacy and safety of troglitazone in the treatment of lipodystrophy syndromes. Ann Intern Med 2000; 133:263-74.

2. Björntorp P. Abdominal obesity and the development of noninsulin-dependent diabetes mellitus. Diabetes Metab Rev 1998; 4:615-22.

3. Bozzette SA, Ake CF, Tam HK, Chang SW, and Louis TA. Cardiovascular and cerebrovascular events in patients treated for human immunodeficiency virus infection. N Engl J Med 2003; 348:702-10.

4. Calmy A, Hirschel B, Hans D, Karsegard VL, and Meier CA. Glitazones in lipodystrophy syndrome induced by highly active antiretroviral therapy. AIDS 2003; 17:770-2.

5. Carr A, Samaras K, Burton S et al. A syndrome of peripheral lipodystrophy, hyperlipidaemia and insulin resistance in patients receiving HIV protease inhibitors. Lancet 1998; 12:F51-F58.

6. Carr A, Samaras K, Thorisdottir A, Kaufmann GR, Chisholm DJ, and Cooper DA. Diagnosis, prediction, and natural course of HIV-1 protease-inhibitor-associated lipodystrophy, hyperlipidaemia, and diabetes mellitus: a cohort study. Lancet 1999; 353:2093-9.

7. Cefalu WT, Wang ZQ, Werbel S et al. Contribution of visceral fat mass to the insulin resistance of aging. Metabolism 1995; 44:954-9.

8. Cohen J. Statistical Power Analysis for the Behavioral Sciences. New York: Academic Press, Inc., 1977.

9. Engelson ES, Glesby MJ, Mendez D et al. Effect of recombinant human growth hormone in the treatment of visceral fat accumulation in HIV infection. J Acquir Immune Defic Syndr 2002; 30:379-91.

10. Engelson ES, Kotler DP, Tan Y et al. Fat distribution in HIV-infected patients reporting truncal enlargement quantified by whole-body magnetic resonance imaging. Am J Clin Nutr 1999; 69:162-9.

11. Ford ES, Giles WH, and Dietz WH. Prevalence of the metabolic syndrome among US adults: findings from the third National Health and Nutrition Examination Survey. JAMA 2002; 287:356-9.

12. Friis-Møller N, Weber R, d'Arminio Monforte A et al. Exposure to HAART is associated with an increased risk of myocardial infarction: the D:A:D study [abstract]. 2003:abstr 130.

13. Fujimoto WY, Bergstrom RW, Leonetti DL, Newell-Morris LL, Shuman WP, and Wahl PW. Metabolic and adipose risk factors for NIDDM and coronary disease in third-generation Japanese-American men and women with impaired glucose tolerance. Diabetologia 1994; 37:524-32.

14. Fujioka S, Matsuzawa Y, Tokunaga K, and Tarui S. Contribution of intra-abdominal fat accumulation to the impairment of glucose and lipid metabolism in human obesity. Metabolism 1987; 36:54-9.

15. Furrer HNQV. Treatment of HAART associated fat accumulation disease witih recombinant human growth hormone: results of a randomized double blind placebo controlled crossover trial. XIII International AIDS Conference, Durban, South Africa, July 9-14, 2000 2000; 2:LbPp114.

16. Gelato MC, Mynarcik DC, Quick JL et al. Improved insulin sensitivity and body fat distribution in HIV-infected patients treated with rosiglitazone: a pilot study. J Acquir Immune Defic Syndr 2002; 31:163-70.

17. Giovannucci E. Insulin-like growth factor-I and binding protein-3 and risk of cancer. Horm Res 1999; 51 Suppl 3:34-41.

18. Glesby MJ. Antiretroviral switching studies--the jury is still out. AIDS Read 2000; 10:482-3.

19. Grunfeld C. Basic science and metabolic disturbances. 2002:Abstr TuOr158.

20. Guan HP, Li Y, Jensen MV, Newgard CB, Steppan CM, and Lazar MA. A futile metabolic cycle activated in adipocytes by antidiabetic agents. Nat Med 2002; 8:1122-8.

21. Hadigan C. A randomized, double-blind, placebo-controlled study of rosiglitazone for patients with HIV lipodystrophy [abstract]. 2003:abstr 12.

22. Hadigan C, Corcoran C, Basgoz N, Davis B, Sax P, and Grinspoon S. Metformin in the treatment of HIV lipodystrophy syndrome: A randomized controlled trial. JAMA 2000; 284:472-7.

23. Hadigan C, Meigs JB, Corcoran C et al. Metabolic abnormalities and cardiovascular disease risk factors in adults with human immunodeficiency virus infection and lipodystrophy. Clin Infect Dis 2001; 32:130-9.

24. Hadigan C, Meigs JB, Rabe J et al. Increased PAI-1 and tPA antigen levels are reduced with metformin therapy in HIV-infected patients with fat redistribution and insulin resistance. J Clin Endocrinol Metab 2001; 86:939-43.

25. Holmberg SD, Moorman AC, Williamson JM et al. Protease inhibitors and cardiovascular outcomes in patients with HIV-1. Lancet 2002; 360:1747-8.

26. Johannsson G, Marin P, Lonn L et al. Growth hormone treatment of abdominally obese men reduces abdominal fat mass, improves glucose and lipoprotein metabolism, and reduces diastolic blood pressure. J Clin Endocrinol Metab 1997; 82:727-34.

27. Kawai T, Takei I, Oguma Y et al. Effects of troglitazone on fat distribution in the treatment of male type 2 diabetes. Metabolism 1999; 48:1102-7.

28. Khan MA, St Peter JV, and Xue JL. A prospective, randomized comparison of the metabolic effects of pioglitazone or rosiglitazone in patients with type 2 diabetes who were previously treated with troglitazone. Diabetes Care 2002; 25:708-11.

29. Klein D, Hurley L, Sorel M, and Sidney S. Do protease inhibitors increase the risk for coronary heart disease among HIV-positive patients? Follow-up through June 2000. Abstracts of the 8th Conference on Retroviruses and Opportunistic Infections, Chicago, IL 2001; abstract 655.

30. Kotler DP, Thompson M, Grunfeld C, Gertner J, Muurahainen N, and on behalf of the STARTS Trial Investigator Group. Transient insulin resistance during recombinant human growth hormone (rhGH) therapy for HIV-associated adipose redistribution syndrome (HARS). 2002:LB-20.

31. Kotler DP, Thompson M, Grunfeld C et al. Growth Hormone (Serostim) effectively reduces viceral adipose tissue (VAT) accumulation and non-HDL cholesterol. abstr LbOr18. 2002.

32. Lamarche B. Abdominal obesity and its metabolic complications: implications for the risk of ischaemic heart disease. Coron Artery Dis 1998; 9:473-81.

33. Lemieux I, Pascot A, Prud'Homme D et al. Elevated C-reactive protein: another component of the atherothrombotic profile of abdominal obesity. Arterioscler Thromb Vasc Biol 2001; 21:961-7.

34. Lo JC, Mulligan K, Noor MA et al. The effects of recombinant human growth hormone on body composition and glucose metabolism in HIV-infected patients with fat accumulation. J Clin Endocrinol Metab 2001; 86:3480-7.

35. Lo JC, Mulligan K, Tai VW, Algren H, and Schambelan M. "Buffalo hump" in men with HIV-1 infection. Lancet 1998; 351:867-70.

35a. Lo JC, Mulligan K, Noor MA, Lee GA, Schwarz, J-M, Grunfeld C, Schambelan M. The effects of low dose growth hormone in HIV-infected men with fat accumulation: a pilot study. Clin Infect Dis 2004; 39:732-5.

36. Martinez E, Conget I, Lozano L, Casamitjana R, and Gatell JM. Reversion of metabolic abnormalities after switching from HIV-1 protease inhibitors to nevirapine. AIDS 1999; 13:805-10.

37. Martinez E, Garcia-Viejo MA, Blanco JL et al. Impact of switching from human immunodeficiency virus type 1 protease inhibitors to efavirenz in successfully treated adults with lipodystrophy. Clin Infect Dis 2000; 31:1266-73.

38. Mary-Krause M, Cotte L, Partisani M, Simon A, and Costagliola D. Impact of treatment with protease inhibitor (PI) on myocardial infarction (MI) occurrence in HIV-infected men. Abstracts of the 8th Conference on Retroviruses and Opportunistic Infections, Chicago, IL 2001; Abstract 657.

39. Mayerson AB, Hundal RS, Dufour S et al. The effects of rosiglitazone on insulin sensitivity, lipolysis, and hepatic and skeletal muscle triglyceride content in patients with type 2 diabetes. Diabetes 2002; 51:797-802.

40. Meigs JB, Mittleman MA, Nathan DM et al. Hyperinsulinemia, hyperglycemia, and impaired hemostasis: the Framingham Offspring Study. JAMA 2000; 283:221-8.

41. Mercie P, Thiebaut R, Lavignolle V et al. Evaluation of cardiovascular risk factors in HIV-1 infected patients using carotid intima-media thickness measurement. Ann Med 1902; 34:55-63.

42. Miller KK, Daly PA, Sentochnik D et al. Pseudo-Cushings's syndrome in human immunodeficiency virus-infected patients. Clin Infect Dis 1998; 27:68-72.

43. Miyazaki Y, Glass L, Triplitt C et al. Effect of rosiglitazone on glucose and non-esterified fatty acid metabolism in Type II diabetic patients. Diabetologia 2001; 44:2210-9.

44. Molarius A and Seidell JC. Selection of anthropometric indicators for classification of abdominal fatness--a critical review. Int J Obes Relat Metab Disord 1998; 22:719-27.

45. Mori Y, Murakawa Y, Okada K et al. Effect of troglitazone on body fat distribution in type 2 diabetic patients. Diabetes Care 1999; 22:908-12.

46. Mulligan K, Grunfeld C, Hellerstein MK, Neese RA, and Schambelan M. Anabolic effects of recombinant human growth hormone in patients with wasting associated with human immunodeficiency virus infection. J Clin Endocrinol Metab 1993; 77:956-62.

47. Mulligan K, Tai VW, and Schambelan M. Effects of chronic growth hormone treatment on energy intake and resting energy metabolism in patients with human immunodeficiency virus-associated wasting--a clinical research center study. J Clin Endocrinol Metab 1998; 83:1542-7.

48. Nakamura T, Funahashi T, Yamashita S et al. Thiazolidinedione derivative improves fat distribution and multiple risk factors in subjects with visceral fat accumulation--double-blind placebo-controlled trial. Diabetes Res Clin Pract 2001; 54:181-90.

49. Nielsen S, Moller N, Christiansen JS, and Jorgensen JO. Pharmacological antilipolysis restores insulin sensitivity during growth hormone exposure. Diabetes 2001; 50:2301-8.

50. Parulkar AA, Pendergrass ML, Granda-Ayala R, Lee TR, and Fonseca VA. Nonhypoglycemic effects of thiazolidinediones. Ann Intern Med 2001; 134:61-71.

51. Pouliot MC, Despres JP, Nadeau A et al. Visceral obesity in men. Associations with glucose tolerance, plasma insulin, and lipoprotein levels. Diabetes 1992; 41:826-34.

52. Roubenoff R, Schmitz H, Bairos L et al. Reduction of abdominal obesity in lipodystrophy associated with human immunodeficiency virus infection by means of diet and exercise: case report and proof of principle. Clin Infect Dis 2002; 34:390-3.

53. Roubenoff R, Weiss L, McDermott A et al. A pilot study of exercise training to reduce trunk fat in adults with HIV-associated fat redistribution. AIDS 1999; 13:1373-5.

54. Safrin S and Grunfeld C. Fat distribution and metabolic changes in patients with HIV infection. AIDS 1999; 13:2493-505.

55. Segerlantz M, Bramnert M, Manhem P, Laurila E, and Groop LC. Inhibition of the rise in FFA by Acipimox partially prevents GH-induced insulin resistance in GH-deficient adults. J Clin Endocrinol Metab 2001; 86:5813-8.

56. Shim M and Cohen P. IGFs and human cancer: implications regarding the risk of growth hormone therapy. Horm Res 1999; 51 Suppl 3:42-51.

57. Sugimoto M, Takeda N, Nakashima K et al. Effects of troglitazone on hepatic and peripheral insulin resistance induced by growth hormone excess in rats. Metabolism 1998; 47:783-7.

58. Sutinen J, Hakkinen AM, Westerbacka J et al. Rosiglitazone in the treatment of HAART-associated lipodystrophy (HAL): a randomized, double-blind, placebo-controlled study. abstr LB13. 2002.

59. Swerdlow AJ, Higgins CD, Adlard P, and Preece MA. Risk of cancer in patients treated with human pituitary growth hormone in the UK, 1959-85: a cohort study. Lancet 2002; 360:273-7.

60. Tien PC, Cole SR, Williams CM et al. Incidence of lipoatrophy and lipohypertrophy in the Women's Interagency HIV Study [abstract 736]. 2003.

61. Torres RA, Unger KW, Cadman JA, and Kassous JY. Recombinant human growth hormone improves truncal adiposity and 'buffalo humps' in HIV-positive patients on HAART [letter]. AIDS 1999; 13:2479-81.

62. Towns R, Kostyo JL, and Colca JR. Pioglitazone inhibits the diabetogenic action of growth hormone, but not its ability to promote growth. Endocrinology 1994; 134:608-13.

63. Walli R, Michl GM, Muhlbayer D, Brinkmann L, and Goebel FD. Effects of troglitazone on insulin sensitivity in HIV-infected patients with protease inhibitor-associated diabetes mellitus. Res Exp Med (Berl) 2000; 199:253-62.

64. Wanke C, Gerrior J, Kantaros J, Coakley E, and Albrecht M. Recombinant human growth hormone improves the fat redistribution syndrome (lipodystrophy) in patients with HIV. AIDS 1999; 13:2099-103.

65. Wilkie S, Chan R, Lees R, Sullivan M, Hadigan C, and Grinspoon S. Increased carotid IMT in women with HIV lipodystrophy. 2001.

66. Yki-Jarvinen H, Sutinen J, Silveira A et al. Regulation of Plasma PAI-1 Concentrations in HAART-Associated Lipodystrophy During Rosiglitazone Therapy. Arterioscler Thromb Vasc Biol 2003; 23:688-94.

67. Zamboni M, Armellini F, Milani MP et al. Evaluation of regional body fat distribution: comparison between W/H ratio and computed tomography in obese women. J Intern Med 1992; 232:341-7.

APPENDIX 1: SCHEDULE OF EVENTS

EVALUATION	SCREEN		ON STUDY EVALUATIONS*						
	- 30 days	**Pre-Entry**	Entry (Day 1)	Week 2	Week 4	Week 6	Week 8	Week 12	Premature Discontinuation (see 6.14)
Informed Consent	Prior to Evaluatio	**Pt teaching**							
Medical and Surgical History	X								
Current Medical Conditions	Until study drug given								
Eligibility Criteria	X								
Randomization			X						
Concomitant Meds/Procedures	Ongoing, throughout study. Query at each visit.								
Adverse Events	Ongoing, after randomization. Query at each visit.								
Physical Exam**	X		X	X	X	X	X	X	X
Anthropometric Measurements	X								
Pregnancy test	X		X[a]	If pregnancy is suspected					
Fasting insulin level	X								
Chemistry Panel (fasting)	X		X	X	X	X	X	X	X
Hematology Panel	X		X	X	X	X	X	X	X
Fasting lipid profile			X		X		X	X	X
HIV viral load, CD4 count			X					X	
HBV, HCV serology			X						
Testosterone level, TSH			X						
Oral Glucose Tolerance Test	X[b]				X[c, e]			X[c,f]	X[c]
FSIVGTT, FFA Flux			X					X	X
Whole Body MRI Scan			X[c]					X	X[d]
DEXA Scan			X					X	X[d]
Bromide, deuterium			X					X	X[d]
REE, food diary, activity quest.			X					X	X
Lipo/mental health quest.			X		X			X	X
HIV Antibody (as needed)	X								
Stored **Serum/**Plasma	X		X		X		X	X	X

* The window for study visits is +/- 1 week

[a] For women of child-bearing potential, a repeat pregnancy test must be done within 7 days of Entry

[b] Send 2 hour glucose level to local lab for determination of subject eligibility

[c] Send 2 hour glucose level to local lab

[d] If after week 4

**Perform complete physical exam at screening & targeted exam thereafter based on symptoms

[e] For Week 4 OGTT: only draw blood at times 0 and 120 mins

[f] Week 12 OGTT should be done within 7 days prior to Week 12 visit

Evaluations for Subjects in Open-Label Phase

EVALUATION	Week 14		Week 18		Week 24	Premature Discontinuation
Eligibility Criteria						
Concomitant Meds/Procedures						
Adverse Events						
Targeted Physical Exam	X		X		X	X
Pregnancy test	If pregnancy suspected					
Chemistry Panel (fasting)	X		X		X	X
Hematology Panel	X		X		X	X
Fasting lipid profile			X		X	X
HIV viral load, CD4 count					X	
Oral Glucose Tolerance Test					X[c]	X[c]
Whole Body MRI Scan					X	X
Stored **Serum/**Plasma			X		X	X

[c] Send 2 hour glucose level to local lab

APPENDIX 2: ORAL GLUCOSE TOLERANCE TEST METHODOLOGY

Perform an Oral Glucose Tolerance Test (OGTT) at the Screening, Weeks 4, 12, and 24 visits as specified in the schedule of events. Follow the procedures specified in the study manual, which are summarized briefly below:

1. Instruct patients to arrive at the site in the morning, following an overnight fast (at least 8 hours).
2. Collect glucose and insulin samples at time zero (Baseline).
3. Administer 75g of glucose ("Glucola") orally at time zero.
4. Collect glucose and insulin samples at 30, 60, 90, and 120 minutes after the patient has finished drinking the "Glucola". **Note: Only collect time 0 and 120 specimens at week 4.**
5. Process and store samples locally at –20 or –70 degrees. Ship to the Cornell GCRC Core Laboratory for analysis at the completion of the study following procedures specified in the study manual.

Screening Visit Only:

Collect an extra sample in a grey top tube at the 120-minute time point. Process and send the sample to the central laboratory for glucose analysis following procedures specified in the study manual. Use the result to assess eligibility according to the criteria in Section 4.1.5, which stipulates that the 120-minute sample from the screening Glucose Tolerance Test must be < 200 mg/dl.

Weeks 4, 12, and 24:

Collect an extra sample in a grey top tube at the 120-minute time point. Process and send the sample to the central laboratory for glucose analysis following procedures specified in the study manual.

APPENDIX 3: ANTHROPOMETRIC MEASUREMENTS

Obtain all anthropometric measurements with a tape measure that is not under tension, using the precise methodology and positioning as described below. Take all measurements in triplicate and enter them into the corresponding Case Report Form (CRF). Calculate the mean values for each measurement and enter them into the CRF.

- Obtain triplicate measures of waist and hip circumferences in a horizontal plane with the study subject standing in a relaxed position.
- The waist circumference is defined as the circumference measured in the horizontal plane at a point immediately below the anterior part of the lowest ribs.
- The hip circumference is defined as the largest circumference measured in the horizontal plane at a point between the iliac crest and the greater trochanter.
- Calculate the waist:hip ratio to determine the study eligibility of each screened subject.

The anthropometric measurements used as eligibility criteria were derived from a consideration of anthropometric and imaging data in the patient and control populations described by Engelson et al [10]. These provide the best available sensitivity and specificity for selection of suitable subjects for this study. Additionally, the waist:hip ratios used as entrance criteria represent those associated with enhanced cardiovascular risk [44].

APPENDIX 4: SAMPLE INFORMED CONSENT FORM

Project Title: **Randomized, Double-Blind, Placebo-Controlled Study of the Safety and Efficacy of Recombinant Human Growth Hormone and/or Rosiglitazone in the Treatment of Human Immunodeficiency Virus-Associated Visceral Adiposity and Insulin Resistance**

Subject: ________________________ **Research Project #** ____________________

Version 3.0 of 02/10/05

PRINCIPAL INVESTIGATOR: PHONE:

INFORMED CONSENT

You are being asked to take part in the research study named above because you are infected with the human immunodeficiency virus (HIV), the virus that causes AIDS, and are thought to have abnormal accumulation of fat in your abdomen called visceral adiposity. To be eligible for the study, you also must have signs of insulin resistance, which means that your body needs to produce higher than usual amounts of the insulin hormone to keep your blood sugar (glucose) within the normal range. Physicians of the _______ hope to learn more about a medication called recombinant human growth hormone (growth hormone, or Serostim) when given with or without rosiglitazone (Avandia) to patients with visceral adiposity and insulin resistance. The study will try to find out the effects of these drugs on insulin/glucose metabolism and abnormal fat accumulation. Before you decide whether or not to take part in this study, we would like to explain its purpose, how it may help, any risks, and what is expected of you. This process is called informed consent.

YOUR PARTICIPATION IS VOLUNTARY

This consent form gives you information about the study, which will be discussed with you. Once you understand the study and if you agree to take part, you will be asked to sign this consent form. You will be given a copy of this form to keep.

Before you learn about the study, it is important that you know the following:

- Your participation is entirely voluntary;
- You may refuse to take part in this study or you may withdraw from this study at any time without losing the benefits of your routine medical care and without any effect on your future care at this hospital.

PURPOSE OF STUDY

Several studies have looked at the effects of growth hormone on abnormal fat accumulation in persons with HIV infection without major problems with insulin resistance. These studies have shown that growth hormone significantly reduces abnormally accumulated deep central fat in the abdomen (sometimes called "visceral adipose tissue") and/or abnormally accumulated fat behind the neck (sometimes called dorsocervical fat or "buffalo hump"). However, a number of HIV-

infected persons with abnormal fat accumulation also have significant insulin resistance. Little is known about the effects of growth hormone in HIV-infected people with insulin resistance. Rosiglitazone is a medication commonly used to treat diabetes that can improve the body's ability to break down (metabolize) glucose in persons who have insulin resistance or diabetes.

The main purpose of this study is to learn how growth hormone, rosiglitazone, or growth hormone plus rosiglitazone affects glucose and insulin levels in HIV-infected patients with insulin resistance compared to no medication at all. The study will also try to learn whether growth hormone with rosiglitazone is safe for HIV-infected people to use for periods up to 24 weeks. It will investigate whether these treatments improve body shape by decreasing abnormal fat accumulation in central parts of the body (abdomen, chest, or back) while producing minimal undesirable loss of fat in the face or limbs. The study will also try to determine whether growth hormone treatment, with or without rosiglitazone, improves levels of fat (lipids) in the blood. Because growth hormone and rosiglitazone may have effects on insulin resistance that are due to their effects on how the body breaks down fat into free fatty acids, we will also study how the body produces free fatty acids during this study.

Rosiglitazone (Avandia) is an oral medication approved by the Food and Drug Administration (FDA) that acts primarily by increasing insulin sensitivity and is indicated to improve control of blood sugars in patients with type 2 diabetes. Growth hormone (Serostim) is an injectable medication approved by the FDA to increase lean body mass and improve metabolism in HIV-infected persons with AIDS wasting (severe weight loss). However, neither rosiglitazone nor growth hormone has been approved by the FDA to reduce abnormal fat accumulation or improve insulin sensitivity in HIV-infected patients with abnormal fat accumulation and insulin resistance.

This study will enroll approximately 80 people and your participation will last for 24 weeks. You may withdraw from the study at any time, and may then choose whether or not you wish to return for follow-up visits with the clinic staff.

PROCEDURES

Screening

If you decide to enroll in this study and sign this consent form, you will be asked about your medical history, the history of your HIV infection, HIV-related events, and non-HIV-related conditions. To be enrolled in the study, you must meet certain pre-specified criteria described in the study protocol. These criteria include, but are not restricted to: (a) having documented HIV infection (b) being between the ages of 18 and 65 years, (c) having evidence of abnormal fat accumulation on physical exam (d) having high insulin levels and/or abnormal blood sugar (glucose) levels after an overnight fast. You must also be taking medications to treat HIV (antiretrovirals) for at least the past 8 weeks, and you must continue to take antiretrovirals for the duration of the study.

In order to determine if you qualify for the study and if it is safe for you to participate, you will have a physical examination and blood tests. The physical exam will include measurement of your waist and hip circumferences (sizes) with a tape measure. About 2 tablespoons of blood will be drawn from a vein in your arm for laboratory tests, including testing the amount of sugar and insulin present in your blood **over a 2 hour period** after you drink a specific amount of

glucose (sugar) and water. The details of this oral glucose tolerance test are described below.~~,~~ ~~**except at the screening visit you will only have blood drawn before and 2 hours (120 minutes) after drinking the sugar solution.**~~ The blood tests must be performed fasting in the morning on an empty stomach (no food or beverages for 12 hours prior to the test). A small amount of blood will also be frozen for future studies of markers for heart disease. If you are a woman who is able to become pregnant, you must have a negative pregnancy test (urine or blood test) at the screening visit and before starting study drug; you will give about 1 teaspoon of blood or a urine sample for the pregnancy test. ~~**You will also be instructed in how to fill out a food diary to record what you have eaten during the 3 days before starting the study. If you qualify for the study, you will be asked to fill out the diary and bring it to the study Entry visit.**~~

During the study, you will be making at least 10 scheduled visits to the study center. (Others may be required if there are problems in scheduling some of the study evaluations or to follow up on abnormal blood test results.) The first visit (the Screening Visit) will be used to determine whether you are eligible to participate in this study. If your medical record does not contain laboratory documentation confirming your HIV infection, a blood sample will be obtained for that purpose. At the Entry Visit(s) additional study evaluations will be performed and you will be given study medication at this visit to start on Day 2. Other follow-up visits will be scheduled for 2, 4, 6, 8, 12, 14, 18, and 24 weeks after study Day 2. The follow-up visits must occur within 1 week of the time that the study protocol says they should occur. Even if you decide to quit the study before the last (Week 24) visit, you will be asked to return to complete the final study evaluations.

Pre-Entry Visit

You will be asked to return to the research clinic for 1 visit before the Entry visit so that you can be taught how to give yourself the injections of the study drug (growth hormone or placebo). At this visit, you will also be instructed to stay on your usual diet until the Entry visit (and for the rest of the study period) and, if you exercise, to try to continue exercising the same amount. You will also be instructed in how to fill out a food diary to record what you have eaten during 3 days before the Entry visit. You will be asked to fill out the diary and bring it to the study Entry visit. If you are a woman who is able to have children, you will be asked to come in for the Pre-Entry visit within 7 days before the Entry visit so that you can give a sample of urine or a teaspoon of blood for a pregnancy test. If you are not able to schedule the Pre-Entry visit within 7 days of Entry, then you may have the pregnancy test done at the Entry visit. The Pre-Entry visit will last approximately 90 minutes.

Entry and Week 12 Visits

The Entry and Week 12 study visits will take place over 2 days and will require one overnight stay in the hospital. You will need to go to St. Luke's-Roosevelt Hospital (113th St. and Amsterdam Avenue) on the morning of the first day to have the following tests done, which are described in detail below: indirect calorimetry, ~~**an oral glucose tolerance test,**~~ an MRI scan, a DEXA scan, and body water measurements. You will have to fast for 12 hours (nothing to eat or drink but water) before these tests. **You should avoid strenuous activity during the evening before these visits, and you should not drink alcohol during the 3 days before these visits. If you have ever had symptoms of alcohol withdrawal (for example, seizures or "DT's") and drink alcohol regularly, please tell the study doctor or nurse immediately since your**

health could be in danger if you suddenly stop drinking alcohol. If you need to take medicine before the tests, take it with sips of water 2 hours before your appointment time. If you have to take your medicine with food, you should wait until after the tests have been completed and the research staff tells you that it is okay to eat. After the tests are done and you have had a chance to eat, you will travel to _______ where you will be admitted to the inpatient General Clinical Research Center part of the hospital. You will be asked general questions about your health and undergo a physical examination. You will also be asked questions about your level of physical activity in an interview that will take about 20 minutes. You will also be asked to turn in the completed food diaries that you were given at the screening and Week 8 visits.

On the morning of the second day of the Entry and Week 12 study visits, you will have the following tests done, which are described in detail below: free fatty acid flux and frequently sampled intravenous glucose tolerance test. You will also have routine blood tests (including blood chemistry, blood counts, cholesterol, T-cell counts, HIV viral load). You will give about 4 tablespoons of blood for the routine blood tests.

During the Entry and Week 12 visits, as well as at the Week 4 visit, you will also be asked to complete about 4 pages of checklists asking you about fatigue, anxiety, depression and confidence about how you look. The questionnaires are checklists about feelings of tiredness, sadness, sleep, appetite, and how you feel about your appearance. If you feel that you may be depressed and you wish to speak to a psychiatrist, an outpatient assessment can be arranged for you. If you feel suicidal, you will be offered an assessment at the emergency room. The questionnaires do not ask for personal information about your history or childhood. The questions may help us to understand whether changes in your body fat are connected to depression and fatigue, and will help us see if changes in appearance cause improvement in mood. You will also be asked to complete a questionnaire at these visits that asks you about changes in the amount of fat in different parts of your body. All together, these questionnaires should take about 30 minutes to complete.

Around the time of the Week 12 visit, you will be asked to return to the research clinic on a separate day for an oral glucose tolerance test (described below). You will need to fast overnight before this visit. The visit will last approximately 2 hours.

Oral Glucose Tolerance Test (OGTT)

You will have an oral glucose tolerance test (OGTT), which is a test that is similar to the test given to pregnant women and others to screen for diabetes. About 2 teaspoons of blood will first be drawn. Then you will be asked to drink a sugary drink that tastes like very sweet soda pop. At 30, 60, 90 and 120 minutes later, about 2 teaspoons of blood will be drawn at each time point (about 4 tablespoons total) to see how well your body handles this sugar. This test takes about 2 hours. **At the Week 4 visit, you will have an OGTT done but blood will only be drawn at the beginning of the test and 120 minutes later.**

Magnetic Resonance Imaging (MRI) scans

Magnetic resonance imaging (MRI) scans will be used to make images and evaluate the composition of your muscle, fat, and bones. MRI scans will be performed at St. Luke's Roosevelt Hospital Center prior to or on Day 1 and at Weeks 12 **and 24** during the study. ~~**About**~~

~~**1 in 4 people in the study (those who are in Group D described below) will also have an MRI scan at week 24.**~~ The MRI scan is performed in an MRI scanner, which is a doughnut-shaped machine. You will be asked to lie down on the MRI scanner table for about 20 minutes during each test while the machine takes pictures of the inside of your body. A small coil resembling a football helmet will be placed over your abdomen or slipped over one of your legs to make special images of your body. You will need to lie quietly without moving during the MRI scan, and you may be asked to hold your breath briefly so that the pictures will not be blurred. You will have to practice holding your breath for almost 30 seconds before you take this test. The MRI table will move you in and out of the scanner, which is shaped like a hollow tube. The MRI scanner will not cause any physical discomfort other than that caused by lying on the table for the duration of the test. The MRI scans are standard procedures, and they do not exposure you to any radiation. The MRI scans won't be analyzed until after the study is over, and these results will not be available to you.

Dual-Energy X-ray Absorptiometry (DEXA) Scans

You will have a whole-body DEXA (dual-energy X-ray absorptiometry) scan 2 times during the study, at the entry visit and week 12. ~~**About 1 in 4 people in the study (those who are in Group D described below) will also have a third DEXA scan at week 24.**~~ DEXA is a special scan that measures body fat, muscle, and bone density. The machine gives off a low amount of radiation that, with the help of a computer, will create a picture of the bones and fat in your body. For a whole-body DEXA, you will lie down on your back on a scanning table for about 5 minutes while an imaging head measuring x-ray transmission moves over your body from head to foot. If you are a woman capable of becoming pregnant, you must have a negative blood pregnancy test within **7 days** before taking this test. The DEXA scans won't be analyzed until after the study is over, and these results will not be available to you.

Body Water Measurements

You will also undergo measurements of the water content of your body at the entry and week 12 visits of the study by 2 different methods. Both of these methods use "tracers", which are very small (trace) amounts of safe substances that you will drink. These tracers will get diluted by the normal water in your body. We will then measure how much of the tracer is left in your blood to get a measurement of how much water is present in your body that diluted the tracer. The first test will measure the total amount of water in your body (total body water). This test requires that you drink approximately 2 teaspoons of deuterium oxide, a non-radioactive tracer. Approximately 1 teaspoon of your blood will be drawn both before and 3 hours after you drink the deuterium oxide. The second test will measure the water in your body that is outside of your cells (extracellular water). The test requires that you drink approximately 1 teaspoon of sodium bromide, a non-radioactive tracer. The test also requires approximately 1 teaspoon of your blood to be drawn before and 3 hours after ~~I~~ **you** drink the bromide.

Free Fatty Acid Flux

The free fatty acid flux test will be performed approximately between 7:00 and 9:00 in the morning, after a 12 hour fast (You will not eat or drink anything but water for 12 hours before the test). While you are lying in bed, an intravenous line (a plastic catheter or flexible plastic tube inserted through a needle) will be placed in a hand vein in order to draw small amounts of blood and after a few minutes a second intravenous line will be placed in your other arm. The

small catheter placed in the vein on the top of your hand will be used to remove samples of blood frequently; this hand will be warmed up with a heating pad to improve blood flow. The other arm will be used for a steady infusion (flow into your vein) of a saline solution (salt in water) containing small amounts of a substance called a stable isotope tracer ([U-^{13}C] palmitate, a fatty acid tracer) and human albumin. The "stable isotope tracer" has no radioactivity. A tracer is administered in very small or “trace” amounts: amounts too small to be noticed compared with the body’s normal rate of metabolism for these compounds. Before administration the fatty acid tracer will have been tested for purity and sterility to be certain that it is free of contaminants capable of producing infection or fever.

You will be lying in bed during the whole test and will only be allowed to sleep, listen to quiet music or watch TV. The test will last about 2 hours and blood will be taken at regular intervals from the hand catheter. About a fourth (1/4) of a cup of blood will be taken.

Frequently Sampled Intravenous Glucose Tolerance Test (FSIVGTT)

The frequently sampled intravenous glucose tolerance test (FSIVGTT) test will be performed at approximately 9:00 in the morning after a 12 hour fast (You will not eat or drink anything but water for 12 hours before the test) and after the “Free Fatty Acid Flux” (see above). While you are lying in bed, an intravenous line will be placed in a hand vein in order to draw small amounts of blood and a second intravenous line will be placed in your other arm. This will be used for the injection of a sterile solution of glucose and, twenty minutes later, for the injection of a very small amount of insulin. Insulin is given to temporarily raise your insulin levels. This drug is of minimal risk (see Risks below). A physician will be available during this procedure to monitor the test. You will need to remain in bed rest during the entire test, but you can read or listen to music.

This test will continue for 3 hours. During this time small samples of your blood will be removed from the small plastic tube in your wrist to measure glucose and insulin. A total of 30 blood samples will be taken over the 3 hours; this will make a total of one half (1/2) cup of blood that will be removed during this test.

Indirect Calorimetry

Your daily calorie needs will be measured by the indirect calorimetry method. This test requires that you lie (in bed) under a clear plastic canopy for about 20 minutes.

Study Treatment

First Study Period (Weeks 0-12):

You will be randomized (assigned by chance, like flipping a coin) to one of the following treatment groups:

Group A: Growth hormone 3 mg injected subcutaneously (under the skin) daily
PLUS Rosiglitazone 4 mg by mouth twice a day

Group B: Growth hormone 3 mg injected subcutaneously (under the skin)
PLUS Rosiglitazone placebo (no active drug) 1 tablet by mouth twice a day

Group C: Weeks 0 to 12: Growth hormone placebo (no active drug) injected subcutaneously (under the skin) daily
PLUS Rosiglitazone 4 mg by mouth twice a day

Group D: Weeks 0 to 12: Growth hormone placebo (no active drug) injected subcutaneously (under the skin) daily
PLUS Rosiglitazone placebo 1 tablet by mouth twice a day

Approximately 20 people will be assigned to each of the study groups. You will have about a 1 in 4 chance of being assigned to each of the groups.

The first part of the study is double-blind, which means that neither you nor your doctor or study nurse will know what drugs you are on. You may not learn what drugs you have been on until after the study is over. In the event of a medical emergency where it is important to know which drugs you have been taking, this information will be provided to your doctor.

Second Study Period:

After completing the first part of the study, including the Week 12 visit, you will move on to the second study period during which you will receive the f ollowing treatments:

Weeks 13-24: Growth hormone 2 mg injected subcutaneously (under the skin) every other day
PLUS Rosiglitazone 4 mg by mouth twice a day

This second part of the study is open-label, which means that you and the study staff will all know that you are receiving the active growth hormone and active rosiglitazone. This part of the study allows everyone in the study, including those who did and did not get active study drugs during the first part, to receive both study drugs for 12 weeks. You will receive a low dose (2 mg every other day) of growth hormone during the second part because the safety of taking the higher dose (3 mg) for more than 12 weeks is not well established. In one small study of 5 patients with HIV infection and no prior growth hormone treatment, 1 mg of growth hormone daily (which is similar to 2 mg every other day) tended to reduce the amount of fat in the abdomen. In a larger study of patients who previously received 2 mg or 4 mg of growth hormone daily for 12 weeks, 2 mg of growth hormone taken every other day was found to be safe and effective at maintaining the loss of fat in the abdomen achieved with the higher dose of growth hormone.

You will be taught how to give yourself the injections ("shots") of research study drug. If you are giving the drug to yourself or if you are having a friend or a family member help you, you must follow the procedures, which the research study doctor or nurse will teach you. All injections should take place in the evening (preferably at bedtime) because this most closely

resembles the way your own body secretes (makes) growth hormone. Rotate the sites of injections between your arms, legs, buttocks and abdomen.

You will be given a container to use for used needles and syringes. Protect others around you from contact with HIV by placing used needles and syringes into this container. Return this disposal container (with the used needles and syringes inside) and other injection supplies to the research study site for appropriate disposal. Keep the research study drug and needles out of the reach of children or persons who cannot read or understand the danger. All research study drugs, which have been dispensed (given) to you (used and unused) must be returned to your physician or nurse. Do not let anyone else take the drug.

The amount of glucose in your blood will be checked regularly to make sure that it is safe for you to continue taking the study medication(s). Your liver function tests and other laboratory values will also be checked for safety. During the course of the study, you may need to reduce the dose or stop taking growth hormone and/or rosiglitazone if your glucose level, liver function tests, other laboratory values become very abnormal, or if you develop undesirable physical side effects. You will be told the most recent results of your laboratory values (blood chemistry tests and blood counts) at each study visit, if you wish to know them. If a blood test shows that certain laboratory values have become very abnormal, you will be contacted and asked to return to the clinic for another blood test at a specified time.

Evaluations On-Treatment (Weeks 0-12):

You will be asked to return to the clinic for follow-up visits at weeks 2, 4, 6, 8, and 12 for a brief physical exam. You will be asked about any HIV-related and non-HIV related symptoms you may have had since your last visit. You will also have about 1 to 2 tablespoons of blood drawn from a vein in your arm for laboratory tests at each of these visits, including testing the amount of glucose present in the blood in the fasting state on some occasions. You may be asked to provide a small cup of urine for lab tests.

At week 4, you will also have an oral glucose tolerance test as described above (Entry and Week 12 Visits) in which an additional **2** tablespoons of blood will be drawn **(right before and 2 hours after drinking the sugar solution, rather than every 30 minutes during the test).**

A small amount of the blood taken at Day 1 and Weeks 4, 8, and 12 will be frozen for future studies. If a blood test shows that any of your laboratory values have become very abnormal, you will be informed and asked to return to the clinic for another blood test at a specified time. If you are a woman who is able to become pregnant, you will have a pregnancy test done whenever pregnancy is suspected; you will give about 1 teaspoon of blood or a urine sample for the pregnancy test.

The Week 12 visit will be similar to the Entry visit and is described above. It will take place over 2 days and will involve an overnight stay in the hospital.

Premature Treatment Discontinuation

If you stop taking the study drug for any reason before week 4, you will be asked to come to the clinic for a follow-up visit that includes an overnight stay and is similar to the Entry visit. You will not need to have the body composition tests (MRI, DEXA, body water measurements). If you stop taking the study drug after week 4, you will be asked to come to the clinic for a follow-up visit that includes an overnight stay and is similar to the Entry visit. You will be asked to have the body composition tests (MRI, DEXA, body water measurements) that would have been scheduled at Week 12.

Evaluations On-Treatment (Weeks 13-24):

After completing the Week 12 visit**,** you will be asked to continue on the study and start low dose growth hormone and rosiglitazone for an additional 12 weeks. If you do not wish to enter the continuation phase, you will be asked to complete the study at the Week 12 visit described above. If you choose to enter the continuation phase, you will return to the clinic for follow-up safety visits at Weeks 14, and 18. You will also have a full day visit at Week 24 (including a visit to St. Luke's-Roosevelt Hospital) that will include an MRI scan and an oral glucose tolerance test.

At each safety visit, you undergo a brief physical examination and you will be asked about any HIV-related and non-HIV related symptoms you may have had since your last visit. You will also have about 1 to 2 tablespoons of blood drawn from a vein in your arm for laboratory tests at each of these visits, including testing the amount of glucose present in the blood in the fasting state on some occasions. At week 24, about 4 additional tablespoons of blood will be drawn as part of the oral glucose tolerance test. You may be asked to provide a small cup of urine for lab tests at some visits. A small amount of the blood taken at Weeks 18 and 24 will be frozen for future studies.

If a blood test shows that any of your laboratory values have become very abnormal, you will be informed and asked to return to the clinic for another blood test at a specified time. If you are a woman who is able to become pregnant, you will have a pregnancy test done whenever pregnancy is suspected; you will give about 1 teaspoon of blood or a urine sample for the pregnancy test.

OTHER INFORMATION

If you have side effects to the study drug, have changes in your laboratory tests, or develop complications of HIV disease, you may be asked to return to the clinic for additional study visits that may require blood to be drawn for laboratory tests.

You may withdraw from this study at any time. If you decide that you wish to withdraw from the study, you must inform the study nurse. You will be asked to complete specific evaluations as described above under "Premature Treatment Discontinuation."

RISKS and/or DISCOMFORTS

The drugs used in this study (Growth Hormone/Rosiglitazone) may have side effects, some of which are listed below. Please note that this list does not include all side effects that might be seen with these drugs but does include the more serious or common side effects with a known, or

possible, relationship. If you have questions concerning the additional study-drug side effects, please ask the medical staff at your site.

The most common side effects reported by subjects receiving rosiglitazone are:

- headache
- edema (swelling) in the legs
- back pain
- anemia (a decrease in the number of red blood cells that may cause weakness, dizziness, and fatigue)
- leukopenia (a decrease in the number of white blood cells that help fight infection)
- hypoglycemia (low blood sugar)
- elevated liver enzymes
- nausea
- vomiting
- abdominal pain
- fatigue
- decreased appetite
- dark urine
- weight gain
- high cholesterol

Side effects less commonly reported with rosiglitazone include fluid accumulation (edema), or worsening of pre-existing fluid accumulation, in the arms and/or legs, in the lungs, and around the heart which may result in breathing problems or congestive heart failure. In particular, congestive heart failure has been reported in some patients with type 2 diabetes who were taking both insulin and rosiglitazone. The most common symptoms of congestive heart failure are edema and shortness of breath. If you experience these symptoms of congestive heart failure, you should immediately contact the study staff. You will be monitored for the development of edema in this study by physical examinations. If edema occurs, you may be asked to eat a low salt diet and to take diuretics ('water pills') if necessary. If you develop symptoms or signs of heart failure, you will be discontinued from the study. Rarely, subjects have developed serious allergic reactions, jaundice, or liver failure.

A drug in the same class as rosiglitazone, called troglitazone, was taken off of the market by the FDA because of cases of liver failure resulting in death or the need for liver transplantation. This type of liver damage has been seen only rarely in patients taking rosiglitazone to date. If you feel any changes after taking this drug, especially unexplained nausea, vomiting, stomach pain, tiredness, loss of appetite, or dark urine, you must immediately contact your personal doctor and the researchers. These symptoms may be the result of changes in the functioning of your liver, one of the possible side effects of rosiglitazone. It is very important that you contact your doctor because there is a chance that liver failure can result, which may be fatal. If your blood tests show that your liver is not functioning adequately, you will be told to stop taking the rosiglitazone. Then you will come weekly for repeat blood tests to monitor your liver function until it returns to normal.

There are additional potential risks associated with rosiglitazone if you are a woman capable of becoming pregnant. In rat and rabbit studies, rosiglitazone caused fetal death and growth retardation before and after birth. Although studies have not been done in pregnant women, rosiglitazone should not be used during pregnancy. Even in women who have not had a period in a long time but are not postmenopausal, pregnancy is possible on rosiglitazone because the drug can cause ovulation (release of an egg from the ovary) to restart. Therefore, you must have a negative pregnancy test before being admitted to the study and must use birth control while on the study and for 3 months after the study ends (see "Pregnancy" section below).

Other side effects that are not commonly known could also occur.

The most common side effects reported by subjects receiving growth hormone are:

- arthralgia (joint or bone pain/discomfort; seen in about 35% of patients)
- edema (swelling; seen in about 35% of patients)
- myalgia (muscle discomfort/pain; seen in about 30% of patients)
- carpal tunnel syndrome (characterized by numbness, tingling, pain, and/or weakness in the hand or forearm)
- nausea
- hyperglycemia (high blood sugar)
- local reaction at the site of injection
- increased blood pressure
- headaches
- enlargement of breast tissue

Usually, undesirable effects are mild and go away either on their own or if the dose of drug is reduced or stopped. Sometimes, undesirable effects can be moderate or severe and require therapy. For example, some patients may require analgesic or anti-inflammatory medicines for joint aches or bone pains.

Regardless of cause, you should report any illness or symptom you have during the research study to your research study doctor. If you have certain undesirable effects (e.g. swelling, joint pain, etc…), your research study doctor may decrease your dose of research study drug by 50% or stop it temporarily. If undesirable effects continue more than seven days after the dose is reduced or research study drug stopped, or if certain severe undesirable effects occur (e.g. carpal tunnel syndrome), your research study doctor may stop your research study drug permanently and remove you from the research study.

Some patients treated with growth hormone have developed glucose intolerance (inability of the body to handle sugar normally) and/or actual diabetes ("sugar"). Some patients needed medication (pills or insulin shots) to treat the diabetes. Some were hospitalized for treatment, and one patient developed diabetic coma (became unconscious because their blood sugar was very high). Please inform the study doctor or nurse if you or anyone in your family (brothers, sisters, parents, grandparents, aunts or uncles) has or has had high blood sugar or diabetes, as this may increase the risk of you developing diabetes while taking growth hormone. Also, please inform the study doctor or nurse as soon as possible if you become very thirsty much of the time, your appetite increases dramatically, you urinate much more than usual, or you become easily

fatigued and sleepy without a good explanation. These symptoms may be signs of high blood sugar or diabetes.

Allergic reactions are possible with growth hormone therapy and prompt medical attention must be sought if an allergic reaction occurs.

Symptoms of allergic reactions can include:
- mild localized swelling
- pain
- redness

However, allergic reactions may also involve the whole body with symptoms such as:
- rash
- itchiness
- swelling
- fever
- palpitations (fast or irregular heart beat)

Or severe allergic reactions (anaphylaxis):
- dizziness,
- loss of consciousness due to very low blood pressure
- difficulty in breathing and swallowing
- abdominal pain
- vomiting
- diarrhea

Prompt medical care is needed since severe allergic reactions are potentially life threatening. If you think you are having an allergic reaction, then recombinant human growth hormone should be stopped and you should call your study doctor immediately or seek emergency medical attention.

Idiopathic Intracranial Hypertension -- increase in fluid pressure in the brain and on the nerve of the eye, leading to symptoms such as headache, nausea, vomiting, visual and hearing problems -- has been reported in children treated with growth hormone for growth hormone deficiency or kidney failure. Some investigators think that contributing risks for this rare syndrome include obesity, high blood pressure, or high fluid overload related to kidney failure. Idiopathic intracranial hypertension is rare and has not been seen in adults treated with growth hormone for AIDS-related weight loss. However, if you develop this condition, you will have to stop taking the drug and you will not be able to continue in the research study. Brain tumor or any other active brain lesion (any condition that affects the brain structures, like a brain abscess or other type of growth) may be a contributing risk for this syndrome. Swelling of an active brain lesion could lead to potentially serious brain problems. If you develop an active brain lesion, you will not be able to continue in the research study.

Growth hormone treatment may or may not cause hypothyroidism (the decreased production of thyroid hormone). In some subjects who have mild thyroid function abnormalities, growth hormone may increase the chance that hypothyroidism may develop. Patients who are being treated for hypothyroidism may need to change their dose of thyroid replacement medication.

Some people who received growth hormone have also experienced pancreatitis, an inflammation of the pancreas, which can be a severe and life-threatening illness, requiring hospitalization for treatment. In some subjects, the cause of pancreatitis was considered related to growth hormone, and in other subjects it was related to infections, other drugs, or other conditions.

Leukemia developed in a small number of children and adults who received growth hormone for multiple reasons. Some had prior leukemia, prior chemotherapy and radiation therapies, or other medical conditions that are known risks for leukemia, and so it is uncertain if growth hormone therapy caused the leukemia. Leukemia was not seen in prior studies of growth hormone in adults with AIDS-related weight loss.

Kaposi's sarcoma, lymphoma (a form of cancer of the lymph nodes), and other malignancies are common in HIV+ individuals. In clinical studies of growth hormone, the risks of developing new Kaposi sarcoma lesions and lymphomas, or progression of cutaneous Kaposi's sarcoma were not found to increase. Patients with internal Kaposi's sarcoma lesions (those inside the body) were excluded from the studies. The potential effects of growth hormone on these Kaposi's sarcoma lesions and on other malignancies are unknown.

There is conflicting evidence about whether growth hormone does or does not increase the occurrence of lymphoma in patients infected with HIV. One analysis suggests that lymphoma might be five times more common, whereas another analysis suggests that lymphoma might be fifty-five times less common in HIV patients given growth hormone. There is also some evidence suggesting the possibility that growth hormone might increase the risk of development of other cancers besides lymphoma, in particular colorectal cancer. Although this evidence is not conclusive, you should discuss with your medical care provider, in case you have known risk factors for this malignancy, the advisability of a regular screening program for colorectal cancer.

Growth hormone should not be taken by a subject with an untreated, active or past cancer except for localized Kaposi's sarcoma of the skin (less than 10 lesions, none of which is greater than about ¾ of an inch in size and not on active therapy). If you are diagnosed with a new or recurrent cancer, you will not be allowed to continue on the research study.

In some laboratory test-tube studies, growth hormone caused the HIV virus to reproduce itself more quickly. When anti-HIV medications such as AZT were added to the experiments, the increased growth of the virus stopped. In prior studies where growth homrone was compared to placebo, no meaningful growth hormone related change in viral load occurred. However, all participants were on anti-HIV therapy during the studies. It is, therefore, important that you remain on your current anti-HIV medications. It is not known whether the anti-HIV medications you are taking will stop the HIV virus from multiplying if your virus is resistant to those medications.

Side effects less commonly or rarely reported in persons receiving growth hormone include fluid accumulation, or worsening of pre-existing fluid accumulation, in the arms and/or legs, in the lungs, and around the heart which may result in breathing problems or heart failure. Rarely, muscle weakness, depression, anxiety, sleep disturbance, hypertriglyceridemia (high levels of a type of fat in the blood called triglycerides), protein in the urine, low platelets (cells that clot the blood), and male breast pain have been reported. Other side effects that are not commonly known could also occur.

Risk related to self-injection:
You may experience temporary discomfort from the daily injections.
These needle sticks may cause:

- local pain (where the needle enters the skin)
- bruising and swelling
- lightheadedness
- dizziness
- rarely, fainting
- local infection

You should change injection sites each day to decrease the chance of local reaction.

Risks of drawing blood
Taking blood may cause some discomfort, bleeding or bruising where the needle enters the body, and in rare cases, fainting or infection.

Risks of Frequently Sampled Intravenous Glucose Tolerance Test
The risks of this procedure are small. The main risk is the occurrence of hypoglycemia (low blood sugar) from the insulin injection. Low blood sugar may produce jitteriness, sweating, headache, dizziness and hunger. However your blood sugar will be monitored constantly during the study and you will be given sugar by vein or by mouth if the blood sugar falls. There are no other side effects with insulin. As with any intravenous injection there is a very small possibility of an allergic reaction occurring; however, the physician in charge of the study could treat this if it occurs. The kind of allergic reactions that ever occur with insulin are very mild (itching all over the body that quickly disappears) and are extremely uncommon (one in 200 people). You may also develop a small bruise at the site of the needles' placements. Also, there is a very small possibility of inflammation or infection at the site, though all precautions will be taken to keep all procedures antiseptic (germ free). When glucose is injected rapidly, some people get flushed, light-headed and have a bad taste in their mouth. This normally goes away in 2 to 3 minutes. Following the test your blood glucose may fall slightly (resulting in mild headache, jitteriness, sweating) but this usually won't last long and will be minimized by eating food provided by the investigators following the test.

Risks of Free Fatty Acid Flux Procedure
The risks of the procedure are small: the needles used for the intravenous line placement may cause pain, bruising, black and blue marks that persist for days, irritation of the vein and a small chance of infection. As with any intravenous infusion there is a very small chance that an allergic reaction may occur. A physician will be nearby at all times and will administer treatment in such a case.

Risks of Body Water Measurements
The only risk is related to the blood draw (see above).

Risk of Oral Glucose Tolerance Test
Some people have nausea and vomiting after drinking the sugar solution.

Risk of MRI Scan

Some people have difficulty when confined to the small space of the MRI scanner and become anxious during the exam. There is no radiation exposure from an MRI scan.

Risk of DEXA Scan

The effective radiation dose from each DEXA study is comparable to 1% of that from a chest X-ray. The biological risk from radiation at this level is considered negligible.

Risk of Indirect Calorimetry

While there are no risks to the indirect calorimetry procedure, some volunteers may be uncomfortable under the canopy. If you are uncomfortable, you can stop the procedure any time.

ADDITIONAL INFORMATION

There is the risk of serious and/or life threatening side effects when non-study medications are taken with study drugs. For your safety, you must tell the study doctor or nurse about all the medications you are taking before you start the study and also before taking any non-study medications while you are on the study.

In addition, you must tell the study doctor or nurse before enrolling in any other clinical trials while you are on this study.

PREGNANCY

Growth hormone and rosiglitazone have not been shown to be absolutely safe to give to pregnant and breast-feeding women. Because of this concern, pregnant and breast-feeding women may not join this study, and women who become pregnant or decide to breast-feed must discontinue the study drug(s). In addition, it is unknown whether the study drug(s) pass through the breast milk and produce bad effects in the infant.

All men and women in this study must agree to use adequate birth control during this study and for 3 months after the study ends. Because it is unknown if the study drugs and/or antiretroviral drug combinations may make birth control pills or depo-progesterone less effective, you should not use them as your only method of birth control. Acceptable barrier birth control methods are a male condom, female condom, or diaphragm. Another acceptable method of birth control is an intra-uterine device (IUD).

If you become pregnant during the study, you should tell your study doctor or nurse right away. Your study drug(s) will be stopped and the study doctor will discuss your choices with you. Your choice will not affect your usual medical care provided by your doctor. You may be allowed to join future research studies, if you qualify.

BENEFITS

Taking part in this study may or may not improve your distribution of body fat or body shape, and no guarantee can be made. You may receive no benefit from this study. However, knowledge gained from this study may in the future help others with HIV infection and AIDS.

You will receive a $20 payment for each completed, routine, scheduled study visit (not including the screening **and pre-entry visits** or extra visits that may be needed due to abnormal blood test results). You will also receive a $200 payment for each completed overnight visit. The total amount of money that you may receive is $5**6**0**.** You will also be reimbursed for travel to and from St. Luke's-Roosevelt Hospital [for Cornell & Columbia sites only] at certain study visits.

NEW FINDINGS

You will be told of any new information learned during the course of the study that might cause you to change your mind about staying in the study. At the end of the study, you will be told when study results may be available and how to learn about them.

REASONS WHY YOU MAY BE WITHDRAWN FROM THE STUDY WITHOUT YOUR CONSENT:

- The investigator decides that continuing in the study would be harmful to you.
- You need to take medications that are not allowed while on this study.
- You are unable to keep appointments or take study drug as instructed.
- You have a bad reaction to study drug(s) or your anti-HIV drugs such that you can no longer continue to take them.
- You become pregnant or breast-feed.
- You no longer meet the eligibility requirements for this study.
- The study is canceled by the Food and Drug Administration or the pharmaceutical companies supplying study treatment.
- Other administrative reasons, such as the discontinuation of the trial at this site.

ALTERNATIVES TO PARTICIPATION

You may choose not to participate in this study at any time. Alternatives to your participation in this study include taking medications by prescription from your health care provider, or you may volunteer for another study if you qualify. Before you decide to take part in this study, your health care provider will give you information about the benefits and risks of the alternative treatments available at your site that may be appropriate for you.

COSTS TO YOU

There is no cost to you for the study treatment, study-related visits, examinations, or laboratory tests in this study. The cost of your anti-HIV drugs, any drugs you may need to treat HIV-related conditions, and any other medical costs for your treatment outside this study, will be charged to you or your insurance company.

CONFIDENTIALITY*(Weill Medical College)*
Your research records will be confidential to the extent permitted by law. You will be identified by a code, and personal information from your records will not be released without your written

permission. You will not be personally identified in any publication about this study. However, your records may be reviewed, under guidelines of the Federal Privacy Act, by the U.S. Food and Drug Administration (FDA), the Office of Human Research Protection (OHRP), Weill Medical College Institutional Review Board (IRB), and study staff.

MEDICAL CARE FOR INJURY RELATED TO THIS STUDY

In accordance with Federal regulations, we are also obliged to inform you about _____'s policy in the event injury occurs. If, as a result of your participation, you experience injury from known or unknown risks of the research procedures as described, immediate medical care and treatment, including hospitalization, if necessary, will be available. However, no monetary compensation is available and you will be responsible for the cost of such medical treatment directly or through your medical insurance and/or other forms of medical coverage. Further information may be obtained by calling _____.

If you have any additional questions regarding this study, please call Dr._____. If you have any questions regarding your rights as a research subject or concerning research-related injury, please call ____.

CONSENT

Please check one of the following boxes to indicate whether or not you wish to have your blood and/or urine stored for IRB-approved, AIDS-related research in the future.

☐ I do wish my blood and/or urine to be stored for IRB-approved AIDS-related research in the future.

☐ I do not wish my blood and/or urine to be stored for IRB-approved AIDS-related research in the future.

If you have read this Informed Consent (or if you have had it explained to you), understand the information, and voluntarily agree to continue participation in this study, please sign your name below.

You will be given a copy of this form to keep.

Signature of Patient or Legal Guardian	Date/Time
Signature of Witness	Date/Time
Signature of Investigator/Co-Investigator	Date/Time

Version 3.0
February 10, 2005
