# Supplementary material for: Recombinant Human Growth Hormone and Rosiglitazone for Abdominal Fat Accumulation in HIV-Infected Patients with Insulin Resistance: A Randomized, Double-Blind, Placebo-Controlled, Factorial Trial
Source: PLoS One. 2013 Apr 12;8(4):e61160. doi: 10.1371/journal.pone.0061160 (PMC3625151; doi:10.1371/journal.pone.0061160)
Supplement: Table S1 — Baseline and Change at Week 12 in Body Weight and Body Composition Parameters by Dual Energy Absorptiometry and Stable Isotope Dilution. (DOC) [file pone.0061160.s004.doc]

Supplementary Table 1: Baseline and Change at Week 12 in Body Weight and Body Composition Parameters by Dual Energy Absorptiometry and Stable Isotope Dilution

| Variable | rhGH/ Rosiglitazone | | Rosiglitazone | | rhGH | | Double Placebo | | P-value* |
| --- | --- | --- | --- | --- | --- | --- | --- | --- | --- |
|  | Entry (n=22) | Change at week 12 (n=21) | Entry (n=19) | Change at week 12 (n=17) | Entry (n=16) | Change at week 12 (n=13) | Entry (n=17) | Change at week 12 (n=15) |  |
|  |  |  |  |  |  |  |  |  |  |
| Weight, kg | 89.4 (20.0) | 1.11 (4.29) | 78.6 (8.58) | 0.71 (1.64) | 92.8 (11.0) | 0.22 (2.39) | 84.0 (17.2) | -0.57 (3.93) | 0.30 |
|  |  |  |  |  |  |  |  |  |  |
| Total fat, kg | 27.0 (11.1) | -2.61^a^ (2.30) | 22.4 (7.00) | 1.14^b^ (1.83) | 29.7 (11.4) | -2.81^c^ (3.16) | 26.7 (8.82) | -0.79^d^ (2.14) | <0.001 |
|  |  |  |  |  |  |  |  |  |  |
| Trunk fat, kg | 17.4 (7.56) | -2.13^d^ (2.00) | 13.9 (3.69) | 0.62^e^ (1.53) | 18.4 (5.79) | -2.38^f^ (2.80) | 16.6 (4.90) | -0.45 (1.40) | <0.001 |
|  |  |  |  |  |  |  |  |  |  |
| Lean body mass, kg | 58.9 (12.8) | 3.54^g^ (3.04) | 53.3 (8.96) | -0.44^h^ (1.59) | 60.4 (9.94) | 2.69^i^ (2.33) | 54.3 (11.4) | 0. 17 (3.30) | <0.001 |
|  |  |  |  |  |  |  |  |  |  |
| Limb fat, kg | 8.72 (4.25) | -0.37^j^ (0.77) | 7.32 (3.56) | 0.49 ^k^ (0.68) | 10.22 (5.96) | ^l^ -0.34 (1.29) | 8.82 (4.22) | -0.32 (0.91) | 0.026 |
|  |  |  |  |  |  |  |  |  |  |
| Total body water, L | 48.3 (10.2) | 1.18 (7.36) | 41.5 (6.22) | 0.22 (2.46) | 45.8 (6.73) | 3.13 (3.69) | 42.5 (8.48) | 0.69 (4.06) | 0.27 |
|  |  |  |  |  |  |  |  |  |  |
| Extracellular water, L | 21.1 (3.56) | 0.38 (4.46) | 18.9 (2.53) | 0.31 (2.23) | 20.6 (3.34) | 0.86 (2.40) | 19.0 (3.86) | 0.27 (3.30) | 0.39 |

Data are expressed as mean (standard deviation). N’s for both total body and extracellular water at entry and week 12 were: rosiglitazone + rhGH n = 21 and n = 20, rosiglitazone n = 18 and n = 17, rhGH n = 15 and n = 12, and double placebo n = 17 and n = 14

*One-way ANCOVA. rhGH x rosiglitazone interaction term was not statistically significant for any variables in two-way ANCOVA with the exception of a trend for limb fat (P = 0.069)

^a^ P = 0.074, ^b^ P = 0.10, ^c^ P = 0.12, ^d^ P = 0.054, ^e^ P = 0.45 ^f^ P = 0.057, ^g^ P < 0.001, ^h^ P = 0.94, ^I^ P = 0.026,

^j^ P = 1.0, ^k^ P = 0.044, ^l^ P = 1.0 compared to double placebo group

Abbreviations: rhGH, recombinant human growth hormone
